# A comprehensive systematic review of human trials investigating herbal treatments for Alzheimer’s disease and dementia

**DOI:** 10.1017/neu.2026.10085

**Published:** 2026-05-08

**Authors:** Katarzyna Aleksandra Kaczmarek-Kryszak, Małgorzata Dobrzyńska, Michalina Banaszak, Sławomira Drzymała-Czyż

**Affiliations:** 1 Department of Bromatology, Poznan University of Medical Sciences, Poland; 2 Doctoral School, Poznan University of Medical Sciences, Poznan, Poland

**Keywords:** cognition, phytotherapy, mild cognitive impairment, vascular dementia, Traditional Chinese Medicine

## Abstract

**Objective:**

Dementia is a group of symptoms, characterised by a loss of cognition that interferes with everyday tasks, difficulty focusing, planning, problem solving, and behavioural changes, such as apathy, anxiety, or depression. The leading cause of dementia is Alzheimer’s disease, but vascular dementia or mild cognitive impairment are also frequently occurring. There are six drugs legislated in Europe for use in the treatment of dementia. There are unmet clinical needs to find more effective, better tolerated or complementary therapeutic options. The aim of this study is to comprehensively analyse the results of clinical trials and other human studies regarding the efficacy and safety of herbal interventions used in patients with dementia.

**Methods:**

We enrolled a total of 48 studies for this systematic review, of which 27 were included into the statistical analysis of effect size (Cohen’s d).

**Results:**

We found significant improvements mainly after administration of Ginkgo biloba, Crocus sativus, Salvia officinalis, and Melissa officinalis. It should be emphasised that some herbs and herbal formulations demonstrated efficacy comparable to that of donepezil, a widely used and approved medication, suggesting potential for phytopharmaceutical therapies as complementary approaches. In some studies, the observed effects were similar to those reported for conventional treatments, indicating promising directions for further research in Alzheimer’s disease and dementia.

**Conclusion:**

In light of the evidence, phytopharmaceuticals have a promising role as a co-therapeutic option or alternative for patients with dementia who do not tolerate or have contraindications to standard medications. However, further research is necessary to translate these initial promising results into clinical practice.

## Graphical abstract

**Figure:**
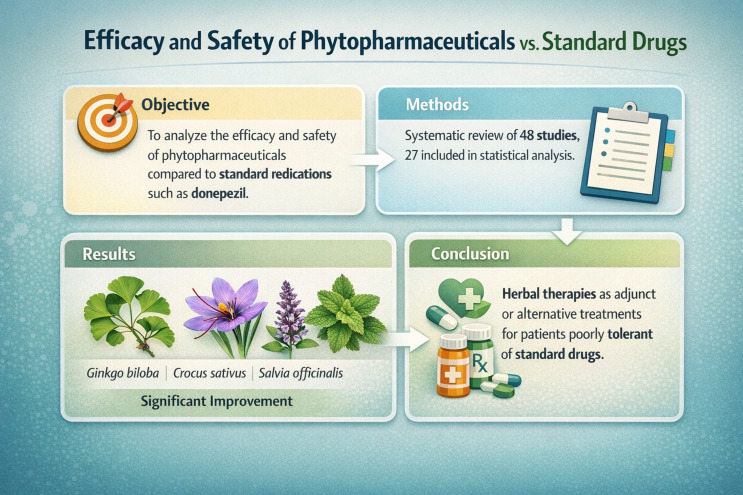

Significant outcomes

- Phytopharmaceuticals have a promising role as a complementary or alternative option for dementia patients who cannot tolerate or respond to standard medications.
- Certain phytopharmaceuticals demonstrated comparable short-term symptomatic effects to standard treatments in small trials; however, evidence is insufficient to support equivalence or superiority.

Limitations

- Many of the studies reviewed are limited by very small sample sizes, which is associated with a high risk of bias when interpreting large effect sizes (Cohen’s *d*).
- The short duration of interventions (often only 3 to 6 months) is insufficient to assess whether phytotherapeutics can constitute disease-modifying treatments (DMTs).

## Introduction

### Sociodemographic implications

The dynamic development of modern medicine has allowed for an increase in the average human lifespan. However, a natural consequence of this phenomenon is demographic ageing; people are living longer and healthier lives, thus the world’s percentage of older people has increased. Statistics for dementia and Alzheimer’s disease, affecting mostly older people (over 65 y.o.), increase hand in hand with the demographic ageing. In 2020 it was estimated that over 55 million people worldwide were suffering from dementia, and it is predicted to double every 20 years (ADI – Dementia statistics, n.d.).

The challenges of Alzheimer’s and dementia goes further than health, and are recognised as an immense socioeconomic burden. Such conditions impact not only the affected individuals but also their families and close communities. The caregivers are exposed to severe distress, as well as social isolation (‘World-Alzheimer-Report-2024.pdf’, no date; no date a). Just in the United States the total costs of Alzheimer’s and dementia patient care was estimated at 384 billion dollars. In Europe those costs vary depending on the country and reach from 163 million EUR to over 32 billion EUR (Jönsson, 2022).

Despite the growing number of clinical trials evaluating natural remedies, a thorough synthesis of the results is necessary to assess their true place in therapeutic regimens for AD, VaD, and MCI.

### Dementia – with emphasis on diagnostic methods

Dementia is not a single health disorder, rather a group of symptoms that can develop on many different origins. The main indicator of dementia is a significant loss of cognition that interferes with everyday tasks, and others include difficulty focusing, planning, problem solving, and behavioural changes, such as apathy, anxiety, or depression (Gale *et al*., 2018; Wilbur, 2023).

The leading cause of dementia is Alzheimer’s disease (AD), a neurodegenerative disorder. The pathological accumulation of amyloid-beta (Aβ) and hyperphosphorylated tau proteins leads to weakening or loss of synapses, and progressive neurodegeneration. In addition, metabolic, vascular, and inflammatory changes, along with other coexisting pathologies, play a crucial role in the development and progression of the disease (Soria Lopez *et al*., 2019).

Vascular dementia (VaD) is the second most common cause of dementia. It encompasses a broad spectrum of cognitive impairments resulting from cerebrovascular disease. These causes include small vessel disease, territorial infarcts, and strategic infarcts. VaD often coexists with AD, leading to mixed dementia (Chang Wong & Chang Chui, 2022).

Between a normal age-related cognitive decline and dementia there is a stage of mild cognitive impairment (MCI). At this stage the patient already presents a decline in cognition but it does not significantly interfere with their daily activities. It can also be an outcome of a different underlying disease or a brain injury (Sanford, 2017; Jongsiriyanyong & Limpawattana, 2018).

To assess the severity of dementia, neurologists and neuroscientists developed many guidelines, including screening tools. The most common is Mini-Mental Status Examination (MMSE), which is used to assess cognitive impairment. It was developed in 1975 and is used to this day. The MMSE is widely applied due to its practicality and short time to run. It consists of 11 questions, covering 5 aspects of cognitive function: orientation, registration, attention and calculation, recall, and language. The maximum score is 30 points, and 23 points or lower indicate a cognitive impairment. Additionally, when used repeatedly, the instrument can track changes in cognitive status that could potentially respond to intervention (Kurlowicz & Wallace, 1999).

The second major tool is the Montreal Cognitive Assessment (MoCA). It is a validated tool used since the 2000, that takes about 10 minutes to complete. It assesses 6 different areas of cognition: short-term memory, visuospatial abilities, executive functions, attention, concentration, and working memory, language, orientation to time and place. The maximum score is 30 points, with the recommendation of applying a 26 point cut-off for cognitive impairment (mocacognition.com, n.d.). Recent comparative studies of MoCA and MMSE have consistently indicated that MoCA exhibits greater diagnostic efficacy than MMSE, mainly due to a better sensitivity (Pinto *et al*., 2019; Siqueira *et al*., 2019; Jia *et al.,* 2021). Nevertheless, both tests are still widely being used.

The Alzheimer’s Disease (AD) Assessment Scale-Cognitive Subscale (ADAS-Cog) is a major tool used for assessing the cognitive deficits in patients with AD. Its main application is for monitoring the efficacy of antidementia treatments. The cognitive subscale of the ADAS-Cog comprises 11 tasks, including both self-administered and observer-based assessments, designed to evaluate functions such as word recall, naming objects and fingers, following commands, constructional and ideational praxis, orientation, word recognition, language abilities, comprehension of spoken language, word-finding difficulties, and remembering test instructions (Kueper *et al.*, n.d.).

Beside rating the cognitive decline, it is important to assess psychological consequences of dementia, which are known as Behavioural and Psychological Symptoms of Dementia (BPSD). The tools used for this rating are: Behaviour Rating Scale for Dementia (BRSD), Behavioural Pathology in Alzheimer’s Disease (BEHAVE-AD), Cohen-Mansfield Agitation Inventory (CMAI), and Neuropsychiatric Inventory (NPI). The BRSD measures a wide range of psychopathology, such as depressive symptoms, psychotic symptoms, aggression, and apathy. The BEHAVE-AD rates paranoia, hallucinations, activity disturbance, aggression, diurnal rhythm disturbances, affective disturbances, and anxiety and phobias. CMAI focuses on agitated behaviours and categorises it into physical, verbal, and aggressive types. The NPI is a very widely used tool, considered to be the ‘golden standard’ for tracking psychiatric changes in dementia patients. It is designed as a caregiver-based assessment that covers delusions, hallucinations, agitation/aggression, apathy, and sleep/night-time disturbances (Tariot *et al.*, 1995; DOMS: Behaviour Measures & Tools, n.d.).

### Approved drugs

Currently in Europe there are six drugs legislated for use in the treatment of dementia. These are three cholinesterase inhibitors (donepezil, galantamine, rivastigmine), an NMDA antagonist (memantine), and two new anti-amyloid therapeutics used for an early stage, mild disorders (lecanemab, donanemab). In clinical trials, donepezil and memantine are the most commonly used positive controls (Dekker *et al*., 2019; Public Health - European Commission, n.d.). Donepezil is a reversible inhibitor of acetylcholinesterase (AChE), an enzyme that normally terminates nerve impulses by hydrolysing acetylcholine (ACh) to choline and acetate. By inhibiting AChE, donepezil prolongs the lifespan of acetylcholine in the synaptic cleft, resulting in increased stimulation of postsynaptic cholinergic receptors. It modifies the pathology of AD by increasing acetylcholine levels in the brain while simultaneously reducing AChE activity. Additionally, donepezil blocks the peripheral anionic site of AChE, preventing Aβ-induced damage to cholinergic neurons and inhibiting the nonclassical activity of AChE, which involves Aβ fibrillation. It also exhibits agonist activity at the sigma-1 receptor (σ1R) (Zhang & Gordon, 2018; Brewster *et al*., 2019).

**Figure 1.**
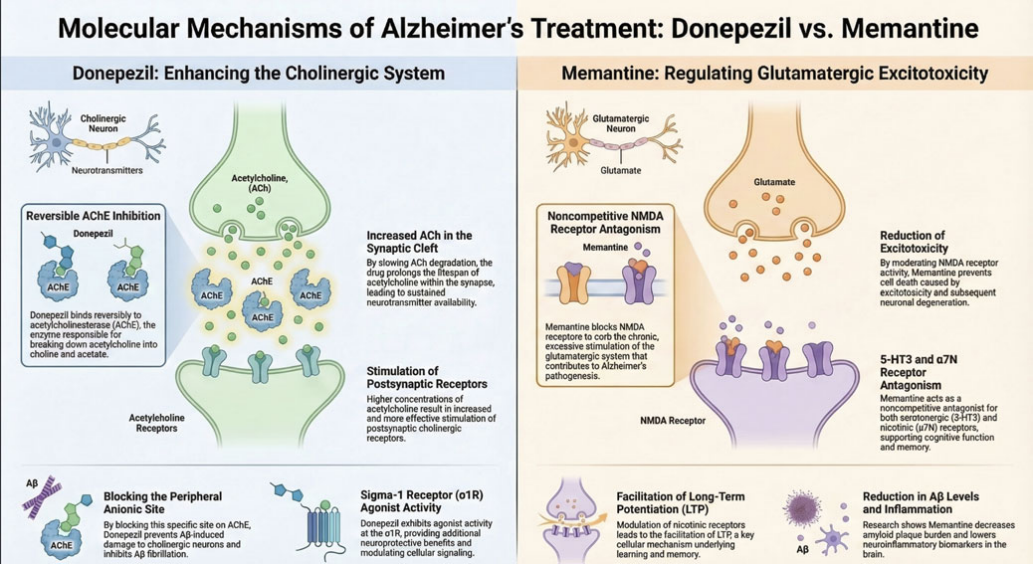
Figure 1 long description. Mechanism of action of donepezil and memantine.

The action mechanism of memantine is primarily based on antagonism of the NMDA (N-methyl-D-aspartate) receptor, as excessive stimulation of the glutamatergic system (with chronic NMDA receptor activity) is thought to contribute to the pathogenesis of Alzheimer’s disease (AD) by inducing excitotoxicity and subsequent neuronal degeneration. Memantine acts as a noncompetitive NMDA receptor antagonist, allowing it to reduce chronic NMDA receptor activity, which leads to cell death. Furthermore, memantine also affects other ion channel receptors belonging to the serotonergic and cholinergic systems, which has a supportive effect on memory and learning. In the serotonin system, it is a noncompetitive 5-HT3 receptor antagonist, which facilitates learning and cognitive functions and may also have antiemetic effects. In the cholinergic system, it is a noncompetitive antagonist of the α7N (nicotinic) receptor, and its action leads to facilitation of LTP (long-term potentiation) and upregulation of nicotinic receptors. In animal models, memantine treatment also resulted in a reduction in Aβ levels and amyloid plaque burden in the brain, as well as a decrease in neuroinflammatory biomarkers (Kishi *et al*., 2017; Balázs *et al.*, 2021). The mechanisms of action of donepezil and memantine are depicted in Figure 1.

Although donepezil and memantine are the first-line drugs for dementia, they are known for many adverse effects. The most common for memantine are agitation, insomnia, confusion, depression, headache, hypertension, dizziness, fall, accidental injury, urinary incontinence, diarrhoea and influenza-like symptoms (McShane *et al*., 2019). Adverse effects of donepezil include diarrhoea, nausea, vomiting, dizziness, and anorexia (Chen *et al*., 2024). Additionally, in the human studies the efficacy of donepezil was comparable to herbal interventions (Mazza *et al.,* 2006; Akhondzadeh *et al*., 2010; Sadhu *et al.,* 2014; Zhang *et al*., 2015; Lin *et al*., 2020; Wang *et al.*, 2020).

There are unmet clinical needs to find more effective, better tolerated or complementary therapeutic options, especially in cases of mild to moderate disease severity. This need is driven by many aspects that require improvement in dementia treatment regimens. These include the numerous side effects of currently used medications, the need to improve the tolerability and safety of drugs, and the limited effectiveness of conventional treatments.

Interestingly, in addition to the pharmacotherapy described above, research is increasingly using plant-derived therapeutics. Herbal preparations are administered in many forms, such as infusions, drops, extracts, or encapsulated powders. In studies different preparations of varying concentrations, obtained using different methods are used. Currently, there are no registered herbal medicines on the pharmaceutical market indicated for the treatment of dementia.

The primary goal of this publication is to comprehensively analyse the results of clinical trials and other human studies regarding the efficacy and safety of natural remedies and complex herbal formulas used in patients with dementia. This review aimed to systematically evaluate the effectiveness and safety of herbal interventions and to compare their clinical effects with standard pharmacological treatments and placebo, including the consistency of outcomes across different types of dementia and the areas of greatest clinical benefit.

## Materials and methods

### Search strategy

Databases Medline (PubMed), Web of Science, Cochrane Library, and Embase were screened for English-language publications concerning phytopharmaceutical interventions in patients suffering from different types of dementia until September 18th, 2025. We used the search terms *‘therapeutic AND plants AND alzheimer*’, *‘therapeutic AND plants AND dementia*’. Additionally, the search criteria were expanded to include the following terms: ‘herbal and Alzheimer’, ‘phytotherapy and Alzheimer’, ‘botanical and Alzheimer’, ‘medicinal plants and Alzheimer’, and ‘traditional medicine and Alzheimer’. It resulted in a total of 3 255 publications. Databases were screened by two independent researchers.

The exclusion criteria were non-research articles (reviews), in-vitro studies, animal studies, computational studies, study protocols, case reports, administration other than oral, studies on healthy subjects, intervention including phytotherapeutic and other treatments (standard drugs, cognitive training, physical activity), study populations with major comorbidities.

**Figure 2.**
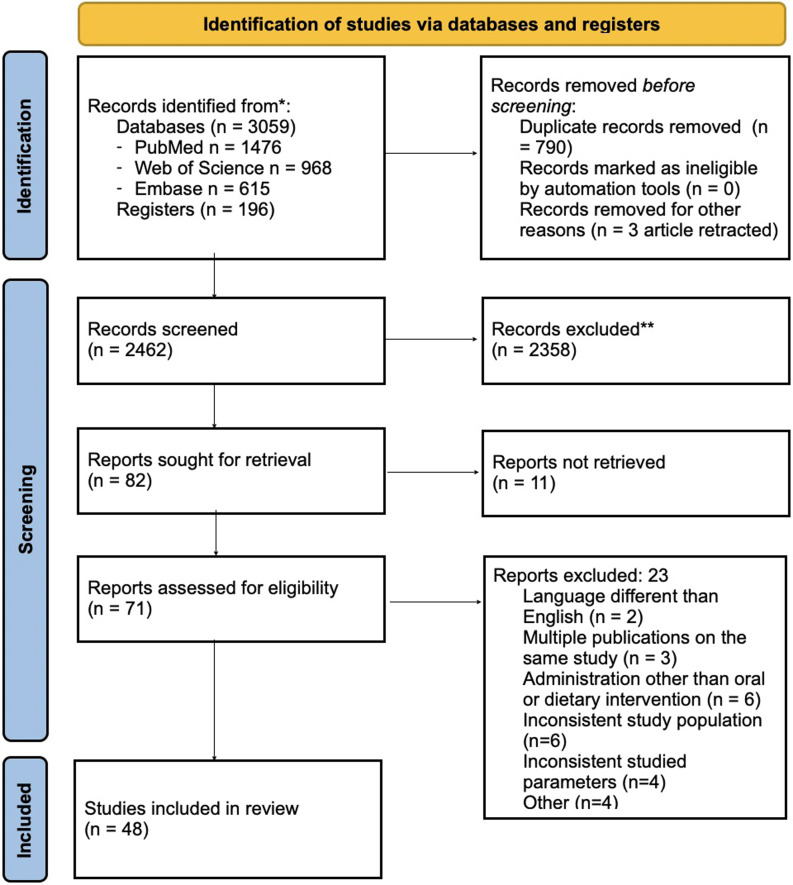
Figure 2 long description. Search scheme.

We included studies on different types of dementia – Alzheimer’s disease, vascular dementia, mild cognitive impairment, and others. We included studies on the intervention with one active substance as well as a mixture or multi-component preparations, intervention with standardised substances (for ex. *Ginkgo biloba* extract EGb 761) or non-standardised (for ex. TCM preparations). Randomised controlled trials (RCTs), double-blind, placebo-controlled studies, and, where necessary, other clinical trials (e.g. open-label, uncontrolled) were included. The search scheme is presented in Figure 2.

The sought data consisted of the type of study, aim, used cognitive tests or scales, results, characteristics of the study group (dementia type, number of patients, sex and age of participants), study duration. In case of missing data on the age range we used mean (SD) given in the publication.

For the risk of bias assessment, two researchers worked independently. We analysed each of the research projects, the presence of a control group/control measurements, the use of validated tests.

### Systematic review protocol

This review was conducted to assess the efficacy of phytotherapeutic interventions in the treatment of different types of dementia. The methodology is based on the PRISMA guidelines and PICO criteria (Leonardo, 2018; Page *et al*., 2021; Nowak & Walkowiak, 2023).

### Qualification criteria (PICO)

Publications meeting the following criteria were included in this study:

#### Population (P)

Studies had to include adult patients diagnosed with cognitive impairment, including:

1. Alzheimer’s disease (AD): Mild, moderate, or severe.
2. Vascular dementia (VaD) or mixed dementia.
3. Mild cognitive impairment (MCI), including vascular MCI and MCI associated with AD.
4. Other dementias (e.g. Lewy body dementia (DLB), Parkinson’s disease dementia (PDD), frontotemporal dementia (FTD)) for which intervention data are available.

#### Intervention (I) and comparison (C)

Studies were included that assessed:

1. Phytopharmaceuticals and TCM: Herbal extracts (e.g. *Crocus sativus, Ginkgo biloba* (EGb 761), *Bacopa monnieri, Cistanche tubulosa*) or complex formulas (e.g. *Jiannao Yizhi Formula, Tiaobu Xinshen Recipe, Yokukansan, Choto-san, Pushen capsule*).
2. Comparison (C): Studies had to include a control group (placebo) or an active comparator (e.g. donepezil, memantine, Ginkgo biloba*). In the absence of a control group, studies had to include paired measurements – before and after the intervention.

* *Ginkgo biloba* was used as a control intervention in some studies, as it is currently the best-researched phytotherapeutic, with relatively predictable and stable outcomes.

#### Outcomes (O)

Key outcome measures for extraction and analysis included:

1. Cognitive function: Changes on the ADAS-cog, MMSE, SKT, and MoCA scales.
2. Functioning of daily living (ADL): Changes on the ADCS-ADL, GBS-ADL, and FAQ scales.
3. Behavioural and psychological symptoms of dementia (BPSD): Changes on the Neuropsychiatric Inventory (NPI) and NPI-Q scales.
4. Safety and tolerability: Incidence of adverse events.

### Statistical analysis

Comparative analysis of the studies was conducted based on *p*-value and effect size (*Cohen’s d*). Effect sizes were calculated based on the numerical values provided in the publication. In the absence of *SD change* values that were necessary to assess *Cohen’s d* a sensitivity analysis was performed to determine this value, using three *r* values; *r* = 0.2, *r* = 0.5, *r* = 0.8. The MMSE and ADAS-cog tests were selected for comparative analysis as the most universal parameter and the ones present in most of the included publications. Calculations were conducted in Numbers (Apple Inc., version 14.4) and Jamovi (version 2.7.13). The eligibility of the studies for the synthesis was assessed by tabulating the interventions and comparing them against the characteristics of the remaining studies. The data we pulled were: phytotherapeutic used as an intervention, dose administered, control type (standard drug/placebo) if applicable, dementia type, used test, number of participants in both groups, *p*-value of the result, mean and standard deviation of the result.

The certainty of the evidence was not formally assessed due to the heterogeneity of study designs and results. However, key factors such as methodological quality and consistency of findings were considered in the narrative synthesis.

#### Risk of bias

Risk of bias assessment was performed independently for each included study. Particular attention was paid to elements such as blinded study design, randomisation, and the presence and nature of the control group (placebo-controlled or nonplacebo-controlled).

#### Statistical synthesis and analysis (methods for calculating effect size)

For all paired comparisons (Intervention vs. Control/Placebo or baseline and endpoint in case of no control group) for which sufficient numerical data (mean, standard deviation, and count) were available, the standardised mean difference (SMD), or Cohen’s effect size (*d*), was calculated.

The formula for pooled standard deviation (SD_pooled_) was used in this calculation to obtain the standardised denominator for the mean difference:

$$d=\frac{M_{A}-M_{C}}{SD_{\text{pooled}}}$$

where:

$$SD_{\text{pooled}}=\sqrt{\frac{(n_{A}-1)SD_{A}^{2}+(n_{C}-1)SD}{n_{A}+n_{C}-2}}$$

A – active group, C – control group

Cohen’s *d* values were interpreted according to conventional thresholds (e.g. *d* = 0.20 small, *d* = 0.50 medium, *d* = 0.80 large). If SD change was needed to be calculated, we used *r* = 0.2, *r* = 0.5, and *r* = 0.8 to show the sensitivity of obtained results. Additionally, we calculated *Cochran’s Q* and *I* ^2^ for partial heterogeneity analysis.

### Heterogeneity analysis and descriptive synthesis

Due to the heterogeneity of the available data, the results that did not qualify for statistical calculations were subjected to descriptive and thematic synthesis.

## Results

We enrolled a total of 48 studies for this systematic review (Tables 1 and 2), of which 27 were included into the statistical analysis of effect size (Table 3). The remaining studies were excluded from statistical analysis due to the use of different diagnostic tests (other than ADAS-cog or MMSE). The statistics also did not include studies with missing necessary numerical data. For plants included in more than one study, we performed heterogeneity analysis (Table 4).

**Table 1.** Descriptive synthesis of single herb interventions Table 1 long description.

Nr	Author	Year	Study type	Aim	Main used test/scale	Result	Conclusion	Study group	N of participants	Sex of participants	Age of participants	Study duration
1	Akhondzadeh S. *et al.*	2010	Randomised, double-blind, controlled study	Evaluation of the effectiveness of *Crocus sativus* in the treatment of mild to moderate AD compared with donepezil.	MMSE, ADAS-cog, CDR	Saffron (30 mg/day) was as effective as donepezil (10 mg/day) treatment. There was a significant improvement in cognition in both groups between endpoint and baseline. There were no significant differences in the ADAS-cog and CDR-SB tests between groups.	*Crocus sativus* supplementation was similarly effective to the donepezil in the case of treating mild to moderate dementia.	Mild to moderate dementia	54 (study group: 27; control group: 27)	29 males; 25 females	55	22 weeks
2	Schneider L. S. *et al.*	2005	Randomised, double-blind, placebo-controlled study	Evaluation of the safety and effectiveness of two different doses of *Ginkgo biloba* extract in elderly patients with probable AD.	ADAS-cog, ADCS-CGIC	There were no significant differences between groups (120 mg GbE, 240 mg GbE, placebo) concerning cognitive decline. There was no significant differences in ADCS-CGIC and ADAS-cog scales.	*Ginkgo biloba* extract did not significantly improve cognitive functions; the lack of effect may be due to slow deterioration in the placebo group.	Dementia or probable AD	513 (study group dose A: 169; study group dose B: 170; control group: 170)	243 males, 270 females	56–98	26 weeks
3	Guo Q *et al.*	2013	Open-label, nonplacebo-controlled, clinical study	Evaluation of the safety and effectiveness of *Cistanche tubulosa* glycosides in the treatment of AD.	MMSE, ADAS-cog, ADL, CGI, Blessed Behavioural Scale	There were no significant differences between baseline and endpoint in the ADAS-cog test, except for recollection command, spoken language, and difficulty in word finding. There were no differences in the MMSE scores. At weeks 36 and 48, there were significant positive changes in personality compared with baseline. There were no significant differences in the ADL assessment. There were no significant changes in overall severity and efficacy between baseline and endpoint.	*Cistanche tubulosa* was not effective in AD treatment; only three ADAS-cog aspects showed improvement.	AD	18	6 males, 12 females	55–80	48 weeks
4	Le Bars P. L. *et al.*	1997	Randomised, double-blinded, placebo-controlled, clinical study	Evaluation of *Ginkgo biloba* extract on the clinical course of dementia of the Alzheimer’s type.	SKT, ADAS, CGI, MWT-B, ZVT	SKT scores improved significantly in the study group, while placebo patients declined. ZVT and ADAS showed slight, non-significant improvement. CGI-Change scores improved significantly in the study group compared with placebo. No EEG differences were found between groups, though the study group improved after 8 weeks.	Data confirmed the clinical efficacy of *Ginkgo biloba* extract in mild to moderate AD.	Mild to moderate dementia of the AD type	18 (study group: 9; control group: 9)	9 males, 9 females	50–80	3 months
5	Farokhnia M. *et al.*	2014	Randomised, double-blind, clinical study	Evaluation of effectiveness and safety of *Crocus sativus* L. stigmas’ extract in preventing decline in moderate to severe AD patients compared to memantine.	SCIRS, FAST, MMSE	There were no statistically significant differences between mean change of SCIRS, FAST, and MMSE scores from baseline to endpoint between study and control group.	*Crocus sativus* L. extract was similarly effective as memantine in reducing cognitive decline; differences were non-significant.	AD	68 (study group:34; control group: 34)	39 males, 29 females	60	12 months
6	Por. *et al.*	1998	Randomised, double-blinded, placebo-controlled, clinical study	Evaluation of the efficacy and safety of *Ginkgo biloba* extract in patients with AD or multi-infarct dementia.	ADAS-cog, GERRI, CGIC	There was no significant change in ADAS-cog scores in the study group, but significantly worse scores in the placebo group. On the GERRI scale, there was slight improvement in the study group and significant worsening in the control group. The CGIC scale showed non-significant worsening in both groups. In ADAS-cog and GERRI scores, mean treatment differences were significantly better for the study group.	Ginkgo supplementation had a minor effect on dementia progression. The outcomes of this study were not sufficient for clinical application.	Dementia in AD or multi-infarct dementia	327 (study group: 166; control group: 161)	No data	45	52 weeks
7	Goswami S. *et al.*	2011	Non-randomised, uncontrolled, prospective, open-label study	Evaluation of the effectiveness of *Bacopa monnieri* on cognitive functions in older-age patients suffering from AD.	MMSE	There was a significant improvement in MMSE scores after drug administration. Significant differences included orientation to time, place, and person, attention, and language. There was no change in registration, recall, or design. Women achieved better scores in orientation, attention, and language compared with men.	Six months of Bacopa supplementation caused a significant improvement in cognition of geriatric patients with AD.	AD	39	15 males, 24 females	60–65	6 months
8	You Y. X. *et al.*	2021	Randomised, double-blind, placebo-controlled study	Evaluation of the effects of *Cosmos caudatus* supplementation in older adults with mild cognitive impairment on cognitive function, mood, biochemical profiles and biomarkers.	MMSE	There was a significant improvement in the study group in the MMSE rating scale, whereas there was no in the control group.	*Cosmos caudatus* supplementation improved global cognition in MCI patients, likely due to polyphenols; also reduced tension and mood disturbance.	MCI	48 (study group: 24; control group: 24)	16 males, 32 females	60–75	12 weeks
9	Scripnikov A. *et al.*	2007	Randomised, double-blind, placebo-controlled study	Evaluation of *Ginkgo biloba* extract EGb 761 on neuropsychiatric symptoms of dementia, as well as caregivers’ distress in comparison to placebo.	SKT, NPI	The composite NPI score and caregiver distress decreased in the EGb761 group and increased in placebo. Clinically significant improvement was noted in 75.3% of the study group and 7.1% of controls. The biggest improvements were in apathy, sleep behaviour, anxiety, irritability, and depression.	*Ginkgo biloba* EGb 761 significantly improved neuropsychiatric symptoms and reduced caregiver distress.	Probable AD, probable AD + cerebrovascular disease or probable cerebrovascular dementia	395 (study group: 98; control group: 197)	11 males, 285 females	50	22 weeks
10	Ihl R. *et al.*	2010	Randomised, double-blind, placebo-controlled study	Evaluation of the efficacy and safety of *Ginkgo biloba* extract EGb 761 in dementia patients, compared to placebo.	DCS-CGIC, DEMQOL-Proxy, ADL-IS, NPI	SKT and ADAS-cog improved significantly in 32% of the study group vs. 15% of placebo. NPI improved in 45% of study patients vs. 24% of placebo. Secondary outcomes (ADL, quality of life, global judgment) also improved consistently.	The intervention improved memory, behaviour, daily functioning, and overall condition vs. placebo, and reduced caregiver distress.	AD, vascular dementia, or mixed form	404 (study group: 202; control group: 202)	132 males, 272 females	50	24 weeks
11	Tsolaki M. *et al.*	2016	Randomised, single-blind, clinical study	Evaluation of the efficacy of *Crocus sativus* L. in the management of symptoms in patients with amnestic and multi domain mild cognitive decline compared to placebo.	MMSE, FRSSD, MoCA, NPI, GDS	MMSE improved in the study group, while controls deteriorated. No differences were found in FRSSD, GDS, or MoCA. MRI showed increased activity in the left temporal lobe; HD-EEG showed better performance in the study group.	*Crocus sativus* supplementation improved temporal lobe activity and EEG parameters, confirming enhanced cognition and information processing.	Amnestic and multi domain mild cognitive decline	35 (study group: 17; control group: 18)	9 males, 26 females	70.57 ± 7.00	12 months
12	Ihl R. *et al.*	2012	Randomised, double-blind, placebo-controlled, double-blind, placebo-controlled study	Evaluation of the efficacy of *Ginkgo biloba* extract EGb 761 on patients with AD and vascular dementia, compared to placebo.	SKT, NPI, ADCS-CGIC	SKT improved slightly across dementia types in the study group, but not in placebo. Significant differences were found in secondary outcomes except quality of life, which improved only in AD patients. SKT improved in 33% of AD and 28% of vascular dementia patients vs. 14% and 19% in placebo. NPI improved in 43% of AD and 54% of vascular dementia patients vs. 22 and 31% in placebo. ADCS-CGIC showed overall improvement in 52% of AD and 64% of vascular dementia patients vs. 26% and 22% in placebo.	The intervention improved cognition in both AD and vascular dementia patients, with similar efficacy and no adverse effects.	Mild to moderate dementia due to probable AD, possible cerebro-vascular disease, or vascular dementia	404 (study group: 139; control group: 133)	132 males, 272 females	50	24 weeks
13	Napryeyenko O. *et al.*	2009	Randomised, double-blind, placebo-controlled study	Evaluation of the efficacy of *Ginkgo biloba* extract EGb 761 by type of dementia in patients with AD and vascular dementia, compared to placebo.	SKT, NPI, HAMD, GBS	Intervention patients improved in all outcomes, while placebo patients deteriorated or remained stable. Responses were similar in AD and vascular dementia, with slight advantage for vascular dementia.	The intervention improved cognition, neuropsychiatric symptoms, and daily functioning compared with placebo.	AD (probable AD or possible AD with cerebrovascular disease) or vascular dementia	391 (study group:196; control group: 195)	110 males, 281 females	50	22 weeks
14	Prabhakar S. *et al.*	2020	Randomised, double-blind, placebo-controlled study	Evaluation of the efficacy of *Bacopa monnieri* in patients with AD or mild cognitive impairment, compared to donepezil.	ADAS-Cog, MMSE, CDR, ADCS-ADL, QOL, COWAT	No differences were found in ADAS-cog change rates. PGIMS improved significantly in the study group vs. controls. No changes were observed in CDR, ADCS-ADL, QOL, naming, word association, or MMSE, except a 3-month MMSE improvement in the donepezil group. Adverse effects included nausea, diarrhoea, asthenia, arthralgia, headache, dizziness, anxiety, restlessness, insomnia, and crying.	The intervention had outcomes similar to donepezil in slowing cognitive decline.	AD or MCI	34 (study group: 17; control group: 17)	22 males, 12 females	50	52 weeks
15	Delfan M. *et al.*	2024	Randomised, triple-blind, placebo-controlled study	Evaluation of the effects of *Bacopa monnieri* on cognitive functions and sleep quality in patients with mild cognitive impairment compared with placebo.	MoCA, PSQI	No differences were found in executive function, orientation, calculation, abstraction, recall, or visuoperception. Attention improved in months 1 and 2, and language in month 2. No differences were found in overall cognition or sleep quality.	*Bacopa monnieri* improved only attention and language; no effect on sleep quality.	MCI	62 (study group: 31; control group: 31)	No data	50	2 months
16	Lee J. *et al.*	2017	Randomised, double-blind, placebo-controlled, pilot study	Evaluation of the efficacy of grape powder formulation on cognitive and regional cerebral metabolism in patients with mild cognitive decline in comparison to placebo.	MMSE, ADAS-cog, HAMD, MFQ, WAIS, BVRT	No significant decline in the study group concerning the standardised volume of interest measures, whereas the placebo group significantly declined in standardised volume of interest of the right posterior cingulate cortex and left superior posterolateral cortex.	The intervention preserved brain metabolism in early AD regions; metabolic stability correlated with attention and working memory.	MCI	10 (study group: 5; control group: 5)	5 males, 5 females	66–82	6 months
17	Ansari M. *et al.*	2025	Randomised, double-blind, placebo-controlled, clinical study	Evaluation of the efficacy of Zataria multiflora on the symptoms of mild to moderate AD compared to placebo.	MMSE, CDR	CDR improved in both groups, but more in the study group. MMSE improved in the study group at baseline, week 4, and week 8; placebo improved at week 4 but declined at week 8.	The intervention led to sustained cognitive improvement and symptom reduction; placebo improvement was transient.	Mild to moderate AD	72 (study group: 36; control group: 36)	30 males, 42 females	50	8 weeks
18	Mazza M. *et al.*	2006	Randomised, double-blind, placebo-controlled study	Evaluation of the efficacy of *Ginkgo biloba* extract EGb 761 in the treatment of mild to moderate dementia of the Alzheimer type, compared to donepezil and placebo	MMSE, SKT, CGI	MMSE showed no change across groups. SKT improved in study and donepezil groups, but declined in placebo. CGI scores improved in study and donepezil groups, with no change in placebo. Study and donepezil groups differed significantly from placebo, but not from each other.	The intervention improved cognition, attention, and memory in AD dementia, similar to donepezil, but better tolerated.	Mild to moderate dementia of the AD type	76 (study group: 25; donepezil group: 25; placebo group: 26)	35 males, 41 females	50–80	24 weeks
19	Lee S. T. *et al.*	2008	Randomised, placebo-controlled, prospective, open-label study	Evaluation of the effects of *Panax ginseng* on cognitive function in patients with AD.	ADAS-cog, MMSE, CDR	After ginseng treatment, ADAS-cog and MMSE scores improved up to 12 weeks. After discontinuation, scores declined to control levels.	*Panax ginseng* was clinically effective in cognitive performance of AD patients.	AD	97 (study group: 58; control group: 39)	33 males, 64 females	45	12 weeks
20	McCarney R. *et al.*	2008	Randomised, double-blind, placebo-controlled, parallel-group, pragmatic study	Evaluation of *Ginkgo biloba* for mild to moderate dementia in a community setting.	NPI, GERRI, ZBI, QOL, EQ-VAS, ADAS-cog	Ginkgo showed no significant effect at six months on ADAS-Cog, QOL-AD.	No evidence that high-purity *Ginkgo biloba* benefits mild–moderate dementia over 6 months.	Mild to moderate dementia	176 (study group: 88; control group: 88)	24 males, 152 females	55	6 months
21	Napryeyenko O. *et al.*	2007	Randomised, double-blind, placebo-controlled, clinical trial, multicentre study	Evaluation of the efficacy of *Ginkgo biloba* EGb 761 in mild to moderate dementia with neuropsychiatric features.	SKT, NPI, ADL, CDT, HAMD	*Ginkgo biloba* EGb 761 improved SKT and was superior to placebo on NPI and ADL. Results were similar in AD and VaD.	Data support safety and efficacy of *Ginkgo biloba*EGb 761 in cognitive and non-cognitive dementia symptoms.	AD or vascular dementia	395 (study group: 198; control group: 197)	110 males, 285 females	50	22 weeks
22	Noguchi-Shinohara M. *et al.*	2020	Randomised, controlled study	Evaluation of the safety and tolerability of rosmarinic acid and its clinical effects and disease‑related biomarker changes.	MMSE, ADAS-cog, DAD, NPI, CDR	No differences in vital signs or neurological exams between *Melissa officinalis* and placebo.	Rosmarinic acid may prevent worsening of neuropsychiatric symptoms in AD.	mild dementia due to AD	23 (study group:12; control group: 11)	12 males, 11 males	60	24 weeks
23	van Dongen M. C. *et al.*	2000	Randomised, double-blind, placebo-controlled, parallel-group, multicentre study	Evaluation of the *Ginkgo biloba* in elderly people with dementia and age-related memory impairment.	NAI, SCAG, GDS, NAI-NAA	No beneficial effects of a higher dose or a prolonged duration of *Ginkgo biloba* treatment.	Ginkgo was not effective in older patients with mild–moderate dementia or age-related memory impairment.	Mild to moderate degree of AD or vascular dementia	214 (study group dose A: 84; study group dose B: 82; control group: 48)	34 males, 180 females	50	24 weeks
24	Akhondzadeh S. *et al.*	2003b	Randomised, parallel group, placebo-controlled study	Evaluation of the efficacy and safety of *Salvia officinalis* extract using a fixed dose in patients with mild to moderate AD.	ADAS-cog, CDR	*Salvia officinalis* extract improved ADAS-cog and CDR-SB at 4 months (*p* = 0.03, *p* < 0.003). Side effects similar, agitation more frequent in placebo.	*Salvia officinalis* extract improved cognition in mild–moderate AD and may reduce agitation.	Mild to moderate AD	39 (study group: 19; control group: 20)	24 males, 15 females	65–80	4 months
25	Akhondzadeh S. *et al.*	2003a	Randomised, double-blind, placebo-controlled study	Evaluation of the efficacy and safety of *Melissa officinalis* extract using a fixed dose in patients with mild to moderate AD.	ADAS-cog, CDR	*Melissa officinalis* improved ADAS-cog and CDR at 4 months (*p* = 0.01, *p* < 0.0001). Agitation more common in placebo.	*Melissa officinalis* extract improved cognition and reduced agitation in mild–moderate AD.	Mild to moderate AD	42 (study group: 21; control group: 21)	24 males, 18 females	65–80	4 months
26	Akhondzadeh S. *et al.*	2009	Randomised, double-blind, placebo-controlled study	Evaluation of the efficacy of saffron in the treatment of mild to moderate AD.	ADAS-cog, CDR	Saffron improved ADAS-cog and CDR at 16 weeks (*p* = 0.04). No differences in adverse events.	Saffron was safe and effective short-term in mild–moderate AD; larger trials are needed.	Mild to moderate AD	46 (study group: 23; control group: 23)	25 males, 21 females	65–80	4 months
27	Albertyn C. P.	2025	Randomised, double-blind, placebo-controlled, feasibility study	Evaluation of the feasibility and safety of nabiximols as a potential treatment for agitation in AD.	CMAI, NPI-NH, QOL, APS, MMSE	No participants withdrew, and adherence was high and was generally feasible to deliver. The intervention was well tolerated.	Nabiximols were feasible and safe for agitation in advanced AD during COVID-19; larger trials are warranted.	Alzheimer disease	29 (study group: 15; control group: 14)	15 males, 14 females	55–95	4 weeks
28	Bäurle P. *et al.*	2009	No-placebo, clinical study	Evaluation of the effectiveness of a traditional Ginkgo fresh plant extract.	DemTect score, SF-12	SF-12 mental score improved. Body and DemTect unchanged. Half reported better memory and concentration; 90% would repeat treatment.	Fresh leaf *Ginkgo biloba* extract is a safe adjuvant option for mild cognitive impairment.	Mild cognitive impairment of the non-AD type	59	15 males, 44 females	60	6 weeks
29	Chowdhury D. *et al.*	2024	Open-label, multicentre, single-arm, phase IV trial	Evaluation of the therapeutic benefits of a defined *Ginkgo biloba* extract in adults with major neurocognitive disorder.	DSM-5, MMSE, TMT, BEHAVE-AD, CGI	At 18 weeks, CERAD, TMT A/B, and BEHAVE-AD improved significantly (*P* < 0.0001). 22% reported adverse events, mostly mild headache/diarrhoea. Two unrelated serious events occurred.	*Ginkgo biloba* EGb 761 showed safety and therapeutic benefits in Indian patients with major neurocognitive disorder.	Major neurocognitive disorder due to AD, major vascular neurocognitive disorder, or mixed forms	150	85 males, 65 females	50	18 weeks
30	Güçer Öz Y. *et al.*	2024	Randomised, pilot-study	Evaluation of the effects of black mulberry (*Morus nigra*) consumption on cognition in patients with mild-to-moderate AD.	MMSE, ADAS-Cog, GDS-15	The control group worsened in MMSE and GDS-15; intervention group stable. ADAS-Cog improved in intervention (*p* = 0.002).	Black mulberry (*Morus nigra*) may slightly improve cognition in AD after 12 weeks.	Mild-to-moderate AD	39 (study group: 20; control group: 19)	23 males, 16 females	65	12 weeks
31	Heo J.H. *et al.*	2012	Open-label study	Evaluation of the effects of ginseng on cognitive function in patients with moderately severe AD.	MMSE, ADAS-cog	High-dose group (4.5 g/day) improved ADAS cognitive/non-cognitive and MMSE at 12 weeks, sustained to 24 weeks.	These results demonstrate the potential efficacy of a heat-processed form of ginseng on cognitive function and behavioural symptoms in patients with moderately severe AD.	Moderately severe AD	40 (study group dose A: 10; study group dose B: 10; study group dose C: 10; control group: 10)	10 males, 30 females	50–90	24 weeks
32	Herrschaft H. *et al.*	2012	Randomised, double-blind, placebo-controlled, multicentre study	Confirmation of the findings of a preceding study (Ihl *et al*., 2011) using the same dosage regimen of EGb 761 in dementia, and further substantiation of clinically relevant treatment effects of the once-daily formulation containing 240 mg of *Ginkgo biloba* extract EGb 761 on cognition and psychopathology in patients with Alzheimer’s disease and vascular dementia, both associated with neuropsychiatric symptoms.	NPI, ADCS-CGIC, ADL-IS, SKT, DEMQOL	*Ginkgo biloba* EGb 761 improved SKT (+2.2 vs. +0.3 placebo) and NPI (+4.6 vs. +2.1 placebo), *P* < 0.001.	*Ginkgo biloba* EGb 761 (240 mg daily) was safe and improved cognition, psychopathology, function, and quality of life.	AD or vascular dementia	410 (study group: 200; control group: 202)	131 males, 278 females	50	24 weeks
33	Kanowski S. *et al.*	1996	Randomised, double-blind, placebo-controlled, multicentre, prospective study	Evaluation of the clinical efficacy of *Ginkgo biloba* special extract EGb 761 in outpatients with dementia of the Alzheimer type and multi-infarct dementia.	MMSE, SKT, MWT-B, CGI	EGb 761 showed significantly more responders vs. placebo. ITT confirmed efficacy in AD and MID.	*Ginkgo biloba* EGb 761 was clinically effective in AD and multi-infarct dementia outpatients.	Presenile and senile primary degenerative dementia of the AD type	156 (study group: 79; control group: 77)	51 males, 105 females	55	24 weeks
34	Kanowski S. *et al.*	2003	Randomised, double-blind, placebo-controlled, fixed-dose, parallel-group study	Evaluation of the clinical efficacy of *Ginkgo biloba* special extract EGb 761 in outpatients with dementia of the Alzheimer type and multi-infarct dementia.	MMSE, ADAS, SKT, CGI, NAB, MADRS	After 24 weeks, EGb 761 improved SKT (–2.1 points) and ADAS-cog (–2.7 points). Placebo showed minimal change (–1.0, –1.3).	Therapeutic effects align with outcomes of another *Ginkgo biloba* EGb 761 study conducted in the U.S.	Presenile and senile primary degenerative dementia of the AD type	205 (study group: 106; control group: 99)	63 males, 142 females	55	24 weeks

**Table 2.** Descriptive synthesis of polyherbal interventions Table 2 long description.

Nr	Author	Year	Study type	Aim	Main used test/scale	Result	Conclusion	Study group	N of participants	Sex of participants	Age of participants	Study duration
1	Wang H. C. *et al.*	2020	Randomised, double-blinded, placebo-controlled, clinical study	Evaluation of the Traditional Chinese Medicine Jiannao Yizhi formula on cognitive functions, and on acetylcholine, tau, and amyloid-β protein 42 levels in the serum of AD patients compared with donepezil.	ADAS-cog, CM-SS, MMSE, MoCA, ADL	ADAS-cog and MMSE scores improved significantly in both groups, without differences at the endpoint. Similarly, CM-SS, MoCA, and ADL scores at the endpoint were not significantly different between groups. However, there was a significant improvement in the study group between baseline and endpoint, and no improvement in the donepezil group.	Jiannao Yizhi Formula was as effective as donepezil for cognition, memory, executive function, and language, and improved serum AD markers.	Probable AD	51 (study group: 27; control group: 24)	8 males, 43 females	50–80	6 months
2	Zhang Y. *et al.*	2015	Randomised, double-blinded, placebo-controlled study	Evaluation of effectiveness and safety of Chinese herbal formula Yishen Huazhuo decoction in improving cognitive function in AD patients in comparison to donepezil.	ADAS-cog, MMSE, NPI, ADL	In ADAS-cog scores, there were no significant differences between groups at weeks 24 and 48. However, the change from baseline to endpoint was significantly different between the study group and the donepezil group. MMSE scores at week 48 were significantly different between groups, as was the mean change from baseline to endpoint. ADL and NPI scores showed no significant differences at any point.	The Yishen Huazhuo decoction improved cognitive functions of AD patients. The effect was superior to donepezil concerning the ADAS-cog test. It also showed less deterioration in the 48th week follow-up compared to donepezil.	Dementia of the AD type	144 (study group:72; control group: 72)	55 males, 89 females	50–85	48 weeks (24 weeks of intervention + follow-up on 48th week)
3	Kimura T. *et al.*	2011	Prospective, open-label study	Evaluation of the effectiveness of ferulic acid and *Angelica archangelica* on behavioural and psychological symptoms of dementia, frontotemporal lobar degeneration, and dementia with Lewy bodies.	NPI, MMSE	There was a significant improvement in NPI scores overall, and in the subscales: delusions, hallucinations, agitation/aggression, anxiety, apathy/indifference, irritability/lability, and aberrant behaviour. Significant improvement was present in both FTLD and DLB groups. Baseline MMSE score was significantly positively correlated with NPI improvement.	The intervention significantly improved patients’ neuropsychiatric symptoms. It was found to be more effective in the treatment of FTDL and DLB symptoms than other.	Frontotemporal lobar degeneration, dementia with Lewy bodies	20	4 males, 16 females	72–92	4 weeks
4	Li S. *et al.*	2019	Randomised, blinded study	Evaluation of the effectiveness and clinical efficacy of the Pushen capsule in the treatment of vascular mild cognitive impairment in comparison to *Ginkgo biloba*.	MMSE, MoCA, SMLRS	There was a significant improvement in the MMSE scores between baseline and endpoint in the study group, but not in the control group. There were no significant differences between groups at the endpoint. There was a significant improvement in the MCi scores in both groups. The study group improved significantly on the SMLRS, but not the control group.	Pushen capsule improved cognition in vascular MCI patients, more effective than *Ginkgo biloba*, especially in memory-related tasks.	Vascular mild cognitive impairment	62 (study group: 30; control group: 32)	35 males, 27 females	18	12 weeks
5	Nagata K. *et al.*	2012	Open-label study	Evaluation and explanation of yokukansan treatment on behavioural and psychological symptoms of dementia in patients with vascular dementia.	MMSE, BI, DAD, UPDRS	TMMSE scores showed no difference between baseline and endpoint. NPI improved overall, especially in agitation and disinhibition. No changes were observed in BI, DAD, or UPDRS. One patient developed pulmonary embolism after deep vein thrombosis.	The intervention improved behavioural and psychological symptoms in vascular dementia, especially agitation and disinhibition, without adverse motor effects.	Vascular dementia	12	9 males, 3 females	61–80	4 weeks
6	Itoh T. *et al.*	1999	Randomised, double-blind, placebo-controlled study	Evaluation of the efficacy of Choto-san on symptoms in patients with vascular dementia.	HDS-R	Intervention was superior to placebo in global improvement, subjective symptoms, and psychiatric symptoms at weeks 8 and 12. Daily living disturbances improved significantly, but neurological symptoms did not.	IThe intervention achieved a good therapeutic efficacy, concerning almost all rated aspects. Choto-san might be an option for the treatment of vascular dementia.	Vascular dementia	139 (study group: 69; control group: 70)	50 males, 89 females	76.6 ± 8.4	12 weeks
7	Yamaguchi S. *et al.*	2003	Open-label, controlled study	Evaluation of the effects of Choto-san on brain activity in patients with stroke-related mild cognitive impairment.	SDS, MMSE	There was a significant improvement in the MMSE score between baseline and endpoint in the intervention group, as well as in the verbal fluency test. There was no change in the SDS score in both groups.	The intervention improved cognition in stroke-related MCI; ERP indicated enhanced neural processing in frontal regions.	Stroke-related mild cognitive impairment	20 (study group: 10; control group: 10)	17 males, 3 females	52–85	12 weeks
8	Lin Z. *et al.*	2020	Randomised study	Evaluation the effects of the Tiaobu Xinshen Recipe in patients with mild cognitive impairment due to AD.	MoCA, MMSE	MMSE and MoCA increased in both groups, but without statistical differences. Dementia syndrome score decreased significantly in the experimental group vs. control.	Tiaobu Xinshen Recipe could effectively improve cognitive impairment of MCI-AD patients.	MCI due to AD	83 (study group: 44; control group: 39)	44 males, 39 females	60–80	12 weeks
9	Ming Z. *et al.*	2023	Randomised, non-inferiority controlled study	Evaluation of the effects of Guilingji capsule in AD.	ADAS-cog, MMSE, CM-SS, ADL	Guilingji capsule improved ‘amnesia’ and ‘slow thinking’ more than ginkgo at weeks 12 and 24.	Guilingji capsule was equivalent to Ginkgo extract in cognition and quality of life.	AD	109 (study group: 58; control group: 51)	Imprecise data	60–85	24 weeks
10	Sadhu A. *et al.*	2014	Randomised, double-blind, placebo-controlled, clinical study	Evaluation of the efficacy of a polyherbal formulation on cognitive functions, inflammatory markers, and oxidative stress in healthy elderly patients as well as those with senile dementia of the Alzheimer type.	MMSE, DDS, FAQ	Twelve-month test formulation improved DSS, immediate recall, and attention span vs. donepezil. FAQ and depression improved; MMSE and delayed recall did not.	Polyherbal formulation showed therapeutic potential in senile dementia of Alzheimer’s type. of Alzheimer’s Type	Healthy elderly subjects and senile dementia of AD	201 (study Alzheimer disease group: 43; study healthy group: 56; donepezil group: 61; placebo group: 41)	No data	60–75	12 months
11	Tajadini H. *et al.*	2015	Randomised, double-blind, placebo-controlled, clinical study	Evaluation of the clinical effectiveness of Davaie Loban in patients with mild to moderate AD.	ADAS-cog, CDR	At 4 and 12 weeks, ADAS-cog scores were significantly better in study vs. placebo. No baseline differences in CDR.	Davaie Loban medication may improve memory in mild to moderate AD.	Mild to moderate AD	44 (study group: 24; control group: 20)	22 males, 22 females	50	12 weeks
12	Iwasaki K. *et al.*	2012	Multicentre, open-label study	Evaluation of the efficacy and safety of the traditional Japanese Kampo medicine yokukan-san for behavioural and psychological symptoms of dementia in patients with dementia with Lewy bodies.	MMSE, DAD, NPI, J-ZBI	Yokukan-san improved NPI and Zarit Burden scores. No MMSE or DAD changes.	Yokukansan improved behavioural and psychological symptoms of dementia with Lewy bodies and reduced caregiver burden without cognitive decline.	Dementia with Lewy bodies	63	30 males, 33 females	55–90	4 weeks

**Table 3.** Effect size comparison Table 3 long description.

Nr	Author	Disorder	Substance	Dose	Test	*p*-Value	Study group mean	Study group SD	control group mean	control group SD	n of SG	n of CG	Cohen’s *d*	conclusion
1	Schneider LS *et al*., 2005	Dementia or probable AD (MMSE 10–24)	EGb 761 vs. placebo	120 mg/day	ADAS-cog	Ns	Mean change: 1.6	SD change: 5.8	Mean change: 0.9	SD change: 5.6	169	174	0.12	EGb761 did not perform better than placebo
				240 mg/day		Ns	Mean change: 1.3	SD change: 5.5	Mean change: 0.9	SD change: 5.6	170	174	0.07	
2	Maurer K *et al*., 1997	Dementia of the Alzheimer type (mild to moderate; BCRS score 3–5)	EGb 761 vs. placebo	240 mg/day	ADAS-cog	Ns	30.33	14.77	36.13	15.56	9	9	−0.38	EGb761 achieved a medium difference, compared to placebo
3	Mazza M *et al*., 2006	Mild to moderate dementia of the Alzheimer type (MMSE 13–25)	EGb 761 vs donepezil	160 mg/day	MMSE	Ns	19.40	3.485	18.55	3.663	25	26	0.24	*G. biloba* effect on the MMSE was similar to both donepezil and placebo
			EGb 761 vs placebo			Ns	19.40	3.485	19.75	3.160	25	25	0.11	
4	McCarney R. *et al.*, 2008	Mild to moderate dementia	*Ginkgo biloba* extract vs. placebo	60 mg × 2/day	ADAS-cog	Ns	20.4	8.2	25	10.3	88	88	−0.49	*G. biloba* gave medium-large effect, compared to placebo
5	Kanowski S. *et al.*, 2003	Presenile and senile primary degenerative dementia of the Alzheimer type (DAT) and multi-infarct dementia (MID)	EGb 761 vs. placebo	240 mg/day	ADAS-cog	0.01	Mean change: −0.85	SD change: 0.68	Mean change: −2.4	SD change: 0.7	20	19	2.25	The use of EGb 761 had a large effect in terms of change in the study group vs. the control group.
6	Akhondzadeh S. *et al*., 2010	Dementia (mild to moderate; MMSE 15–26)	*Crocus sativus*’ stigmas extract vs. donepezil	15 mg/day	ADAS-cog	Ns	Mean change: −3.96	SD change: 3.5	Mean change: −3.77	SD change: 3.8	24	23	−0.05	*C. sativus* was similarly efficient as donepezil
7	Tsolaki M *et al*., 2016	Amnestic and multi domain mild cognitive decline	*Crocus sativus* L.	–	MMSE	0.015	28.18	1.24	27.11	2.82	17	18	0.49	*C. sativus* gave a medium effect on the MMSE scale compared to the control group
8	Akhondzadeh *et al*., 2010	Mild to moderate Alzheimer’s disease	*Crocus sativus* vs. placebo	15 mg × 2/day	ADAS-cog	0.001	Mean change: −3.69	SD change: 1.69	Mean change: 4.08	SD change: 1.34	20	20	−5.09	The effect of *C. sativus* was very large, compared to placebo
9	Goswami S *et al*., 2011	AD	Standardised *Bacopa monnieri* extract (Bacognize®)	300 mg × 2/day	MMSE	<0.001	Baseline: 19.15; endpoint: 23.85	Baseline: 4.54; endpoint: 7.61	–	–	39	–	*r* = 0.2, *d* = 0.58; *r* = 0.5, *d* = 0.71, *r* = 0.8, *d* = 0.97	Bacognize administration gave a large difference on the MMSE scale
10	Sadhu A. et al., 2014	Senile Dementia of Alzheimer’s Type	*Bacopa monnieri*, Hip pophae rhamnoides, and *Dioscorea bulbifera* vs. donepezil	500 mg	MMSE	Ns	7.882	1.956	7.914	2.106	43	61	−0.02	This polyherbal formulation gave an effect similar to donepezil
11	Noguchi-Shinohara M. et al., 2020	Mild dementia due to Alzheimer’s disease	*Melissa officinalis* extract containing rosmarinic acid vs. placebo	500 mg/day	MMSE	Ns	Mean change: −2.10	SD change: 3.73	Mean change: −0.40	SD change: 2.27	10	10	−0.55	*M. officinalis* showed a medium effect on the MMSE compared to placebo, but none on the ADAS-cog
					ADAS-cog	Ns	mean change: 2.40	SD change: 5.50	Mean change: 1.60	SD change: 5.32	10	10	0.15	
12	Akhondzadeh S. et al., 2003a	Mild to moderate Alzheimer’s disease	*Melissa officinalis* extract vs. placebo	60 drops/day (∼3 ml)	ADAS-cog	0.001	Mean change: −6.40	SD change: 1.66	Mean change: 5.6	SD change: 1.40	20	15	−7.72	The effect of *M. officinalis* was very large, compared to placebo
13	Nagata K *et al*., 2012	Vascular dementia	Yokukansan	2.5 g/day	MMSE	Ns	Baseline: 15.3; endpoint: 15.8	baseline: 8.5; endpoint: 9.0	–	–	12	–	*r* = 0.2, *d* = 0.05; *r* = 0.5, *d* = 0.06, *r* = 0.8, *d* = 0.09	Yokukansan achieved no effect on the MMSE scale
14	Iwasaki K. *et al*., 2012	Dementia with Lewy bodies.	Yokukansan extract	2.5 g YKS powder × 3/day	MMSE	Ns	Baseline: 18.0; endpoint: 19.2	baseline: 7.0; endpoint: 7.4	–	–	23	–	*r* = 0.2, *d* = 0.13; *r* = 0.5, *d* = 0.17, *r* = 0.8, *d* = 0.26	There was a small size effect concerning the impact of YKS on the MMSE scale ratings
15	Guo Q *et al*., 2013	AD (MMSE 10–18)	*Cistanche tubulosa* glycosides	300 mg × 3/day	ADAS-cog	Ns	Baseline: 34.77; endpoint: 34.37	baseline: 6.38; endpoint: 6.84	–	–	18	–	*r* = 0.2, d = −0.05; *r* = 0.5, d = −0.06, *r* = 0.8, d = −0.1	*C. tubulosa* did not achieve significant effect in the ADAS-cog scale change
16	Wang HC *et al*., 2020	Probable AD (MMSE 10–26)	Jiannao Yizhi Formula vs, donepezil	5 g × 2/day	ADAS-cog	Ns	17	07.04	17.63	6.23	27	24	−0.1	The effect of Jiannao Yizhi Formula was similar to donepezil on the ADAS-cog scale, but not on the MMSE.
					MMSE	Ns	24.33	2.27	25.13	2.6	27	24	−0.33	
17	Zhang Y *et al*., 2015	Dementia of the Alzheimer type	Yishen Huazhuo decoction vs. donepezil	100 ml/day	ADAS-cog	<0.05	19.65	10.62	25.16	11.21	72	72	−0.51	Yishen Huazhuo decoction had small difference on the MMSE scale, compared to donepezil, and medium effect on the ADAS-cog
					MMSE	<0.05	22.37	5.31	20.6	4.52	72	72	0.36	
18	You YX *et al*., 2021	Mild cognitive decline	*Cosmos caudatus* powder capsule vs. placebo	500 mg/day	MMSE	0.049	Mean change 1.82	SD change: 1.19	Mean change: 0.8	SD change: 1.32	23	24	0.81	*C. caudatus* achieved a large effect on the MMSE scale, compared to placebo
19	Yamaguchi S *et al*., 2004	Post-stroke mild cognitive impairment	Choto-san vs. placebo	7.5 g/day	MMSE	<0.05	Mean change: 1.4	SD change: 5.4	Mean change: 0.2	SD change: 3.8	10	10	0.26	Choto-san showed a small effect on the MMSE rating change compared to placebo
20	Lee J *et al*., 2017	Mild cognitive decline	*Vitis vinifera* powder formulation vs. placebo	36 g × 2/day	ADAS-cog	0.06	Mean change: 1.13	SD change: 3.09	Mean change: −2.58	SD change: 1.8	5	5	1.47	*V. vinifera* achieved a very large effect on the ADAS-cog scale, but not on the MMSE, compared to placebo
					MMSE	Ns	Mean change: 1.2	SD change: 2.39	Mean change: 1.2	SD change: 1.64	5	5	0	
21	Ansari M *et al*., 2025	Mild to moderate AD	Zataria multiflora vs. placebo	600 mg × 3/day	MMSE	<0.0001	24.28	2.65	19.83	2.91	36	36	1.6	*Z. multiflora* achieved a very large effect on the MMSE rating, compared to placebo
22	Lee S. T. *et al*., 2008	Alzheimer Disease	*Panax ginseng powder vs. placebo*	4.5 g/day	ADAS-Cog	0.407	Mean change: −3.5	SD change: 4.2	Mean change: −2.8	SD change: 4.2	58	39	−0.17	*P. ginseng* did not show a difference in the effect compared to placebo.
					MMSE	0.673	Mean change: 0.56	SD change: 3,6	Mean change: 0,88	SD change: 2.5	58	39	−0.10	
23	Lin Z. *et al*., 2020	Mild cognitive impairment caused by Alzheimer’s disease	Tiaobu Xinshen Recipe vs. donepezil	15 mL × 2/day	MMSE	<0.05	27.41	1.85	27.62	1.71	44	39	−0.12	The effect of Tiaobu Xinshen Recipe was similar to donepezil
24	Ming Z. *et al*., 2023	Mild-to-moderate probable Alzheimer disease	Guilingji capsule vs *Ginkgo biloba*	2 capsules/day	MMSE	0.209	24.3	1,6	23.3	2.7	60	60	0.45	Guilingji capsule achieved a medium effect on the MMSE ratings, but small on the ADAS-cog, compared to *G. biloba*
					ADAS-Cog	0.423	13,3	8,7	16,4	12	60	60	−0.30	
25	Akhondzadeh S. *et al.*, 2003b	Mild to moderate Alzheimer’s disease	*Salvia officinalis extract vs. placebo*	60 drops/day (∼3 ml)	ADAS-cog	0.001	Mean change: −6.60	SD change: 1.63	Mean change: 5.53	SD change: 1.12	15	15	−8.67	The effect of *S. officinalis* was very large, compared to placebo
26	Güçer Öz Y. *et al.*, 2024	Mild-to-moderate AD	*Morus nigra* (black mulberry concentrate) vs. no intervention	20 g/day	MMSE	Ns	Mean change: −0.85	SD change: 0.68	Mean change: −2.4	SD change: 0.70	20	19	2.247	The effect of intervention vs. placebo was very big, yet the change in the study group alone was non-significant
					ADAS-cog	0.002	Mean change: −4.6	SD change: 1.29	Mean change: −2.4	SD change: 1.94	20	19	−1.34	The effect size of mulberry vs. no intervention was very big, and the intervention effect alone was significant
27	Heo J.H. *et al*., 2012	Moderately severe Alzheimer’s disease	Sun ginseng (SG)-135 vs. no intervention	1.5 g/day	MMSE	Unstated	14.4	8.7	20.00	4.5	10	10	−0.81	The biggest effect size on both scales was achieved in the 3g per day group, yet only the 4,5g per day group had a significant difference between baseline and endpoint.
					ADAS-cog	Unstated	29.0	20.8	26.1	11.6	10	10	0.17	
				3 g/day	MMSE	Unstated	13.7	4.7	20.00	4.5	10	10	−1.37	
					ADAS-cog	Unstated	35.1	15.7	26.1	11.6	10	10	0.65	
				4.5 g/day	MMSE	Unstated	17.00	8.2	20.00	4.5	10	10	−0.45	
					ADAS-cog	Unstated	28.5	23.3	26.1	11.6	10	10	0.13	

**Table 4.** Partial heterogeneity analysis Table 4 long description.

Plant vs. placebo	Number of studies	*Cochran’s Q*	*I* ^2^(%)	Interpretation
*Ginkgo biloba*	6	42.84 (*p* < 0.001)	88.3	The statistics indicate very high heterogeneity. The main source of inconsistency is the Kanowski study (*d* = 2.25, *n* = 39), whose results drastically differ from the large Schneider studies (*d* = 0.12, *n* = 343 and 0.07, *n* = 344), which failed to demonstrate superiority over placebo.
*Crocus sativus*	2	56.31 (*p* < 0.001)	98.2	Extreme heterogeneity was noted. This results from the dramatic difference between the moderate effect in the more recent Tsolaki study (*d* = 0.49, *n* = 35) and the extremely strong effect reported by Akhondzadeh (*d* = −5.09, *n* = 40). This heterogeneity suggests that these results cannot be directly combined without paying attention to differences in protocols.
*Melissa officinalis*	2	42.52 (*p* < 0.001)	97.6	As with *Crocus sativus*, there is a significant degree of inconsistency. Although both studies suggest Melissa’s effectiveness, the magnitude of this effect in the Akhondzadeh study (*d* = −7.72, *n* = 35) is incomparably larger than in the more recent sample (*d* = −0.55, *n* = 20), making it statistically impossible to consider them as consistent evidence.

The most examined herbal therapeutic used in dementia and other neuropsychiatric disorders is *Ginkgo biloba*, especially the EGb 761® agent. EGb 761 is a standardised dry extract derived from *Ginkgo biloba* leaves, containing 22.0–27.0% ginkgo flavonoids and 5.0–7.0% terpene lactones. The latter comprise 2.8–3.4% ginkgolides A, B, and C, and 2.6–3.2% bilobalide. The extract contains less than 5 ppm of ginkgolic acids (Kandiah *et al*., 2019). The effects of GBE are pleiotropic and have been confirmed in numerous *in vitro* and *in vivo* models. GBE has the ability to modify cerebral blood flow, strengthen capillary walls, and prevent blood clot formation (Smith *et al*., 1996; Diamond *et al*., 2000; Maclennan *et al*., 2002; Ahlemeyer & Krieglstein, 2003). *G. biloba* extract protects cultured neurons from death induced by hypoxia, glutamate, cyanide, and amyloid-*β*. It has been shown to improve energy metabolism, stabilise mitochondrial membrane potential, and regulate pro-and antiapoptotic proteins, thereby reducing cell death following oxidative stress (Ponto & Schultz, 2003; Smith & Luo, 2004; Shi *et al*., 2009). Oxidative stress imbalance is a hallmark of AD pathogenesis. *G. biloba* extract acts as a free radical scavenger, mitigating reactive oxygen species (ROS) levels. Additionally, it can increase the activity of endogenous antioxidant enzymes such as superoxide dismutase (SOD) and catalase, and enhance glutathione (GSH) synthesis. The flavonoid fraction is primarily responsible for antioxidant activity (Colak *et al*., 1998; Wei *et al*., 2000; Bridi *et al.,* 2001). *G. biloba* extract may protectprotects against Aβ-induced neurotoxicity, which has been linked to the inhibition of Aβ production by lowering free cholesterol levels, Aβ oligomerisation, and iron-chelating properties, which may inhibit Aβ fibril formation (Bastianetto *et al*., 2000; Yao *et al*., 2004; Ramassamy, 2006; Shi *et al*., 2009).

Most studies have focused on assessing the efficacy of standardised *Ginkgo biloba* extract (EGb 761) in Alzheimer’s dementia (AD), vascular dementia (VaD), or mixed forms. Multiple randomised controlled trials (RCTs) have shown significant improvements in SKT scores in the EGb 761 group, while a decrease in brain performance was observed in the placebo group (Le Bars *et al*., 1997; Kanowski & Hoerr, 2003; Scripnikov *et al*., 2007; Herrschaft *et al*., 2012). The biggest studies (*n* > 400) reported improvements in the ADAS-cog and SKT scores in 32% of subjects taking EGb 761, compared to 15% in the placebo group (Ihl & Bachinskaya, 2010). In the study by Herrschaft et al., patients treated with EGb 761 improved their SKT scores by 2.2 ± 3.5 points, while the change in the placebo group was only 0.3 ± 3.7 points (*p* < 0.001) (Herrschaft *et al*. 2012). These differences were consistent across subgroups, demonstrating similar efficacy in AD and vascular dementia (Napryeyenko *et al.*, 2007; Napryeyenko *et al*., 2009; Ihl *et al*., 2012). In one study, the percentage of patients with clinically significant improvement in the ADCS-CGIC was 52% in the AD group and 64% in the VaD group (Ihl *et al*., 2012).

Some large RCTs found no significant differences in cognitive decline on the ADAS-cog between the *Ginkgo biloba* groups and placebo (van Dongen *et al.,* 2000; Schneider *et al*., 2005; McCarney *et al*., 2008). A 2008 study by McCarney et al. found no significant effect of EGb 761 on the ADAS-cog score (McCarney *et al*., 2008). Furthermore, a 2000 study by van Dongen found that Ginkgo was not effective in treating mild to moderate dementia (van Dongen *et al*., 2000).

Treatment with *Ginkgo biloba* EGb 761 significantly improved neuropsychiatric symptoms and reduced caregiver distress compared to placebo (Scripnikov *et al*., 2007; Napryeyenko *et al.,* 2009; Ihl & Bachinskaya, 2010). A study from 2012 showed that the Neuropsychiatric Inventory (NPI) composite score improved by 4.6 ± 7.1 in the *Ginkgo biloba* EGb 761 group, compared to 2.1 ± 6.5 in the placebo group (*p* < 0.001) (Herrschaft *et al*., 2012). Significant improvements were also observed in all secondary outcome measures, including daily living (ADL) and the Clinical Global Assessment (CGI) (Napryeyenko *et al.,* 2009; Ihl & Bachinskaya, 2010; Herrschaft *et al*., 2012).

Another interesting and well-studied plant species in dementia is *Crocus sativus.* Its therapeutic effects are attributed to its main bioactive components: crocin, crocetin and safranal (Hatziagapiou *et al*., 2019). Saffron and its components are powerful antioxidants. The main carotenoids in saffron (crocin and crocetin) reduce reactive oxygen species (ROS), thus protecting nerve cells from damage (Avgerinos *et al*., 2020; D’Onofrio *et al*., 2021). Some studies suggest that aqueous methanolic extracts of saffron can moderate AChE activity, which increases acetylcholine concentrations in synapses and thereby improves cholinergic transmission and cognitive function. AChE inhibition is primarily attributed to safranal, as well as crocetin and dimethylcrocetin (Avgerinos *et al*., 2020). Saffron extracts and crocetin exhibit high affinity for the phencyclidine binding site of the NMDA receptor. This modulation may lead to an antagonistic effect similar to memantine, helping to regulate glutamate levels and prevent excessive excitation of nerve cells, which leads to their death (Berger *et al*., 2011; Geromichalos *et al*., 2012). crocin and crocetin, exhibit antiaggregatory effects on the Aβ peptide. Crocetin stabilises Aβ oligomers (the most toxic form) and prevents their transformation into neurotoxic fibrils. Crocin may prevent Aβ aggregation by binding to the carotene core, stabilising the central, aggregation-prone hydrophobic cluster, thus preventing the transition from an *α*-helix to a neurotoxic β-sheet structure (Papandreou *et al*., 2006; Ahn *et al*., 2011; Ghahghaei *et al*., 2013). Crocin also inhibits the aggregation of human tau protein (by reducing the ratio of *β*-sheet structure to loose/random structure), which is important in the context of neurofibrillary tangle (NFT) formation, the second pathological hallmark of AD (Zarei Jaliani *et al*., 2013; Karakani *et al*., 2015; Hire *et al*., 2017).

Studies comparing saffron with standard treatments and placebo indicate a potential therapeutic effect. Data from short‑term trials suggest that saffron may provide cognitive benefits comparable to those observed with donepezil in patients with mild to moderate Alzheimer’s disease. In a 16‑week double‑blind, placebo‑controlled study, saffron (30 mg/day) showed similar short‑term improvements in ADAS‑cog and CDR‑SB scores to those seen with donepezil (10 mg/day), with no significant differences between groups. However, the study’s short duration and small sample size limit the strength of these findings and do not allow conclusions regarding long‑term efficacy. Saffron extract demonstrated similar efficacy to memantine in reducing cognitive decline in patients with moderate to severe AD, with no statistically significant differences in the SCIRS, FAST, or MMSE scales (Farokhnia *et al*., 2014a). In another study, saffron resulted in significantly better cognitive scores compared to placebo (ADAS-cog: *p* = 0.04; CDR: *p* = 0.04) after 16 weeks (Akhondzadeh *et al*., 2010). In patients with amnestic MCI, saffron supplementation resulted in significant improvements in MMSE scores (*p* = 0.015) (Tsolaki *et al*., 2016). Although these findings suggest potential cognitive benefits, the available studies are short in duration and involve relatively small samples, limiting conclusions regarding long‑term efficacy.

The plant that has been a subject of extensive research is *Bacopa monnieri.* It exhibits potent neuroprotective and antioxidant effects, primarily attributed to bacosites. *B. monnieri* prevents neuroinflammation and restores redox balance (i.e. the balance of oxidation and reduction) in the brain, mitigating oxidative stress. It does this by modulating inflammatory pathways (e.g. the TNF and IL-6 pathways) and by strengthening the internal defence system, including restoring reduced glutathione (GSH) levels and increasing the activity of antioxidant enzymes (SOD, CAT, GPx) (Preethi *et al*., 2012; Liu *et al*., 2013; Maruthiyodan *et al*., 2026). *B. monnieri* extracts protect neurons from amyloid-beta (Aβ)-induced neurotoxicity and even reduce Aβ levels in AD mouse models (Aguiar & Borowski, 2013). The action of *B. monnieri* involves activation of choline transferase (ChAT), which leads to increased synthesis of acetylcholine (ACh). Furthermore, it increases dendritic branching (arborisation) in neurons, which is associated with improved learning and neuronal plasticity (Vollala *et al*., 2011; Maruthiyodan *et al*., 2026).

Studies included in this review, comparing *Bacopa monnieri* with donepezil, found no significant difference in the rate of deterioration on the ADAS-cog over 52 weeks (Prabhakar *et al*., 2020). The herbal formula containing *B. monnieri* produced an effect similar to donepezil on the MMSE (Sadhu *et al*., 2014). In a study of patients with MCI, *B. monnieri* showed improvement only in the attention and language subscales (Delfan *et al*., 2024). In an uncontrolled study of patients with AD, significant improvements were noted on the MMSE after six months, with the greatest changes in orientation and language abilities (Goswami *et al*., 2011).

Other herbs tested for therapeutic properties against dementia are *Panax ginseng*, *Melissa officinalis*, *Salvia officinalis*, or *Cosmos caudatus*, among other plants emerging in phytopharmaceutical studies. *Melissa officinalis* and *Salvia officinalis* have a beneficial effect on dementia, mainly through their ability to modulate the acetylcholine signalling pathway through an acetylcholine receptor activity. Each of those herbs display binding properties with both nicotinic and muscarinic receptors, while *Melissa officinalis* also compensates for neurotransmitter deficits through an inhibition of acetylcholine esterase (AChE) (Akhondzadeh *et al*., 2003a, 2003b). Ginsenosides of *Panax ginseng* reduce Aβ40 and Aβ42 levels, regenerate axons and synapses, and minimise the inhibitory effect of Aβ on cholinergic transmission in the hippocampus. They also act as antioxidants, increase synaptic density in the hippocampus, and enhance the proliferation and differentiation of neural progenitor cells (Lee *et al*., 2008). Other herbs, such as *Cosmos caudatus* are characterised by a high amount of flavonoids. *C. caudatus* contains quercetin and quercitrin. These chemical compounds have powerful free radical scavenging properties. They potentially reduce oxidative damage to DNA and lipids and prevent lipid peroxidation (You *et al.*, 2021b).

These herbs also had variable results in dementia patients. *Melissa officinalis* and *Salvia officinalis* reduced agitation in Alzheimer’s subjects (Akhondzadeh *et al*., 2003a, 2003b). *Panax ginseng* turned out to be a transient cognitive enhancer, yet there was no difference compared to placebo (Lee *et al*., 2008). *Cosmos caudatus* achieved a large effect on MMSE scale (*d* = 0.81) attributed to high polyphenol concentration (You *et al.*, 2021b). *Zataria multiflora* gave significant and sustained improvements in cognitive function (MMSE, *d* = 1.6) and dementia symptoms compared with placebo, which was transient. In this study a large effect on MMSE was observed in mild to moderate AD (Ansari *et al*., 2025). On the contrary, *Cistanche tubulosa* was not effective in the treatment of AD. No significant differences were found between baseline and endpoint in the ADAS-cog and MMSE (Guo *et al*., 2013).

Traditional Chinese Medicine (TCM) preparations are becoming increasingly popular in dementia research. Blends such as Choto-san, *Yokukansan, Jiannao Yizhi Formula, Yishen Huazhuo decoction, Pushen capsule, Tiaobu Xinshen Recipe, or Guilingji capsule* are complex herbal mixtures developed based on the principles of energy balance and harmonisation of organ function. In the context of treating dementia, including Alzheimer’s disease, they demonstrate multifaceted biological effects.

Experimental and clinical studies have shown that these preparations may exhibit neuroprotective, anti-inflammatory, antioxidant, and modulatory effects on the cholinergic and glutamatergic systems, as well as inhibiting β-amyloid aggregation and tau protein phosphorylation. Some of them also improve cerebral blood flow and metabolism, which may translate into beneficial effects on cognitive function (Itoh *et al*., 1999; Iwasaki *et al*., 2012; Zhang *et al*., 2015; Li *et al*., 2019; Lin *et al*., 2020; Wang *et al*., 2020; Ming *et al*., 2023). Despite promising preliminary research results, clinical evidence regarding the efficacy and safety of these preparations remains limited, with most data coming from studies with small sample sizes and varying methodological quality. Therefore, TCM preparations are currently viewed as potential adjunctive therapies requiring further validation in well-designed, randomised clinical trials.

Yishen Huazhuo decoction showed greater improvements than donepezil on the ADAS‑cog and MMSE scales. The MMSE score also improved more markedly in the herbal treatment group. However, these studies did not include a placebo control group and were limited to short‑term observation periods (24 weeks), which restricts the ability to draw firm conclusions about the true magnitude of the effect and its long‑term clinical relevance. (Zhang *et al*., 2015). The intervention with *Choto-san* was significantly superior to placebo in terms of global improvement, subjective symptoms, and impairment in daily functioning in vascular dementia (Itoh *et al*., 1999). In the post-stroke MCI the effect was primarily on symptoms and functioning, with a small effect on change in the MMSE (*d* = 0.26) (Yamaguchi *et al*., 2004).

The optimal doses of selected plant extracts used in interventions according to the source studies are presented in Table 5. Additionally, we included the mechanism of action of each of them.

**Table 5.** Optimal doses of selected plant extracts and their mechanism of action Table 5 long description.

Plant	Optimal dose	Mechanism of action
*Ginkgo biloba*	240 mg × 1 per day	Antioxidant Iron-chelation GSH synthesis enhancement
*Crocus sativus*	15 mg × 2 per day	Antioxidant AChE inhibition NMDA antagonist Aβ and tau antiaggregation
*Bacopa monnieri*	300 mg × 2 per day	Antioxidant ChAT activation Neuronal arborisation increment
*Melissa officinalis*	∼3 ml × 1 per day	ACh pathway modulation AChE inhibition
*Salvia officinalis*	∼3 ml × 1 per day	ACh pathway modulation
*Panax ginseng*	4.5g × 1 per day	Aβ antiagregation Axones and synapses generation NPCs proliferation and differentiation
*Cosmos caudatus*	500 mg × 1 per day	Antioxidant

## Discussion and conclusions

The main purpose of this study was to assess the effects of phytopharmaceutics on cognition and neuropsychiatric symptoms in dementia patients. Most of the studied plants, herbs and formulas had a great positive clinical impact on treating mild to moderate dementia, especially *Ginkgo biloba, Crocus sativus*, and *Salvia officinalis*, which supports the hypothesis of their therapeutic value (Akhondzadeh *et al*., 2003b, 2010; Ihl & Bachinskaya, 2010; Ihl *et al*., 2012; Tsolaki *et al*. 2016).

Although *Ginkgo biloba* showed results of a large effect size, the outcomes were not consistent throughout all of the studies. Some authors obtained significant improvement of EGb 761 interventions in the field of SKT, ADL, and NPI (Kanowski *et al*., 1996; Le Bars *et al*., 1997; Kanowski & Hoerr, 2003; Mazza *et al*., 2006; Scripnikov *et al*., 2007; Napryeyenko *et al*., 2009; Ihl & Bachinskaya, 2010; Ihl *et al.,* 2012; Herrschaft *et al*., 2012; Chowdhury *et al.,* 2024), whereas some did not achieve any difference between *Ginkgo biloba* and placebo (Por & Evans, 1998; van Dongen *et al*., 2000; Schneider *et al*., 2005; McCarney *et al*., 2008). These inconsistencies might be influenced by an abnormal deterioration rate in the placebo group, like in the case of studies by Schneider *et al.* (Schneider *et al*., 2005). The other reason might be the differences in test sensitivity. In most cases, the effects measured by the SKT score were significantly better, whereas the same intervention did not show any differences on other scales. This happened in the study by Le Bars *et al.* on *Ginkgo biloba* efficacy, a significant improvement on the SKT scale was noted, yet the measurements on ADAS were negligible (Le Bars *et al*., 1997). Similar outcomes were revealed by Mazza *et al.* where the SKT improved significantly, while MMSE did not show any changes (Mazza *et al*., 2006). The differences in the results suggest that the SKT scale may have been more sensitive to changes compared to the ADAS-Cog or MMSE scales in the studied populations, which creates a bias in the interpretation of the results. However, there was a consistent effectiveness of EGb761 on the neuropsychiatric symptoms. NPI scores improved in the studies of Ihl *et al.*, Herrschaft *et al.* and Scripnikov *et al.* (Scripnikov *et al*., 2007; Ihl and Bachinskaya, 2010; Herrschaft *et al*., 2012).

*Crocus sativus* extract has been repeatedly demonstrated to be comparable to conventional medications. Studies have shown that saffron (at a dose of 30 mg/day) was as effective as donepezil (10 mg/day) in the treatment of mild to moderate AD. In these clinical trials, no statistically significant differences were observed in ADAS-cog and CDR-SB scores between the saffron and donepezil groups (Akhondzadeh *et al*., 2010). Despite indications of potential cognitive benefits, the currently available studies are short-term and include relatively small samples, which limits the ability to draw conclusions about long-term efficacy. Furthermore, saffron demonstrated similar efficacy to memantine in reducing cognitive decline in patients with moderate to severe AD, with no statistically significant differences on the SCIRS, FAST, and MMSE scales (Farokhnia *et al*., 2014a).

Similar results were observed for complex TCM formulas. The *Jiannao Yizhi formula* proved equally effective as donepezil in terms of cognitive function, memory, executive function, and language skills. In this case, scores on the ADAS-cog and MMSE significantly improved in both groups, but the differences at the end of the study were not significant (Guo *et al*., 2013). Similar efficacy in slowing cognitive decline, comparable to donepezil, was also observed in a study evaluating a complex preparation containing, among others, *Bacopa monnieri* extract (Sadhu *et al*., 2014).

The *Yishen Huazhuo decoction* demonstrated superiority over donepezil in improving ADAS-cog scores. The change in ADAS-cog scores between baseline and post-baseline was significantly different in favour of the herbal formula, and achieved a medium effect size (*d*=−0.51) (Zhang *et al*., 2015). A comparative study suggested that *Pushen capsule* – a formula consisting mainly of *Polygonum multiflorum, Pollen Typhae, Salvia miltiorrhiza, Ligusticum chuanxiong*, and paeoniflorin – may have superior beneficial effects over *Ginkgo biloba* extract in vascular MCI. *Pushen capsule* improved cognitive function, especially in the field of memory (Li *et al*., 2019). Wang *et al.* in a meta-analysis from 2021, showed a promising potential in numerous Chinese herb formulas. One of them was *Bushen Jianpi Huatan Pills* (formula containing *Cistanche deserticola, Rhizoma Alismatis, Polygonum multiflorum thumb*), which scored significantly higher compared to placebo in the MMSE scale. It also showed an optimistic effect of the *Guipi Decoction* (mixture consisting of 14 herbs), combined with donepezil and nimodipine (versus both drugs alone) on the MMSE. The last one, which achieved a significant effect was the *Dream sweet oral liquid* (17 herbs) with citicoline, compared to the citicoline alone (Wang *et al*., 2021).

In addition to stabilising cognitive function, a key therapeutic goal in dementia treatment is managing behavioural and psychological symptoms of dementia (BPSD), such as apathy, anxiety, agitation, and depression. These symptoms significantly lower patients’ quality of life and increase caregiver distress. *Ginkgo biloba* extract (EGb 761®) is well documented in this context, demonstrating significant improvements in the NPI score and reducing caregiver stress (Scripnikov *et al.,* 2007; Ihl *et al*., 2012; Kandiah *et al*., 2019). Other phytopharmaceuticals also play a significant role in mood and behaviour modulation. In clinical trials, *Melissa officinalis* and *Salvia officinalis* demonstrated a positive effect not only on improving cognitive function (ADAS-cog) but also on reducing agitation (Akhondzadeh *et al*., 2003a, 2003b). These findings are consistent with other authors’ reports. Noguchi-Shinohara et al. described *Melissa’s* properties in the treatment of AD, through anti-A*β* action, cholinergic pathway modulation, and both anti-inflammatory and anti-oxidative characteristics. Participants received the intervention via aromatherapy (Noguchi-Shinohara *et al*., 2020). Kennedy et al. used encapsulated dried leaves of *Melissa* and achieved a great improvement in patient’s calmness and cognition (Kennedy *et al*., 2003). Burns et al. also utilised aromatherapy as a form of *Melissa’s* delivery, and reached similar effects to donepezil concerning, agitation and NPI scores (Burns *et al*., 2011). Regarding sage, Lopresti described its wide-range anti-oxidant and anti-inflammatory properties, which were confirmed in numerous in vitro and animal studies (Lopresti, 2017). Kennedy *et al.* studied *Salvia’s* impact on mood in AD patients and found it effective in reducing anxiety, improving calmness, alertness, and task performance (Kennedy *et al*., 2006). Other combination formulations, such as *Yokukansan*, have shown significant improvement in behavioural and psychological symptoms (BPSD) in dementia with Lewy bodies (DLB), including delusions, hallucinations, and aggression/agitation, without compromising cognitive function (Nagata *et al*., 2012). In a study of *Cosmos caudatus* in elderly individuals with MCI, significant improvements in tension and total mood disturbance were observed (You *et al.*, 2021b).

Although the largest evidence base concerns *Ginkgo biloba* and *Crocus sativus*, this review indicates the presence of other, less-researched substances that have demonstrated exceptionally large clinical effect sizes in smaller trials, requiring further clinical research. *Zataria multiflora* achieved a very large effect size (*d* = 1.6) on the MMSE scale in patients with mild to moderate AD compared to placebo. The benefits of *Zataria* on brain health were already proven in animal studies. Arab et al. used a rat model of AD and revealed that it reversed the disorders caused by lipopolysaccharide in the Morris maze and dark chamber tests. It also lowered the oxidative stress through increasing the levels of superoxide dismutase, catalase, and IL-6 (Arab *et al*., 2022). Eskandari-Roozbahani et al. however, did not reveal any changes in the oxidative levels in rat’s brain tissue. Nevertheless, they reported improvement in the Morris maze test, reduction of AChE and increased levels of Brain-Derived Neurotrophic Factor (BDNF) (Eskandari-Roozbahani *et al*., 2019).

Another plant to achieve a large effect size (*d* = 0.81) on the MMSE scale in patients with MCI was *Cosmos caudatus*, which is attributed to its high concentration of polyphenols, such as quercetin and quercitrin. You et al. performed an interesting study on Task-Based Dorsolateral Prefrontal Cortex Activation through an MRI imaging. They administered 500 mg *C. caudatus* capsules, twice a day for 12 weeks to older adults with MCI. They revealed that this intervention significantly improved the digit span test scores, working memory test scores, and Stroop test score, which implicates a better cognitive control and information processing (You *et al.*, 2021a).

Many of the beneficial effects of phytopharmaceuticals go beyond the anticholinergic mechanism of action, which is the one modulated by conventional drugs. Oxidative stress is a key factor in the pathogenesis of AD. Studies clearly indicate that antioxidant properties are important. *Cosmos caudatus*, for example, significantly reduced serum malondialdehyde (MDA) concentrations and increased glutathione (GSH) concentrations (You *et al.*, 2021b). Similarly, *Bacopa monnieri* prevented oxidative stress by restoring redox balance and increasing the activity of antioxidant enzymes such as SOD, catalase, and glutathione peroxidase (Aguiar & Borowski, 2013). Action against *β*-amyloid peptide (Aβ) and tau protein aggregation is another key mechanism. In the case of *Crocus sativus*, crocin and crocetin have been shown to protect against Aβ-induced toxicity. Crocin inhibits tau protein aggregation and the production of Aβ oligomers (Hatziagapiou *et al*., 2019). EGb 761 has been shown to protect neurons against Aβ-induced cell death and inhibit Aβ aggregation (Singh *et al.,* 2019; Lorca *et al*., 2023). Some substances act as transient cognitive enhancers. In the case of *Panax ginseng*, the observed improvement in the ADAS-cog and MMSE scales disappeared after treatment discontinuation, suggesting that its effect is rather the result of neurotransmitter modulation (dopamine and serotonin) than modification of the disease pathology (Lee *et al*., 2008).

The great advantage of phytotherapeutic interventions is their good tolerability and high safety profile. Studies have shown that *Melissa officinalis* was well-tolerated and did not cause safety issues (Noguchi-Shinohara *et al*., 2020). Furthermore, phytotherapeutic agents often outperform conventional medications in terms of safety. In clinical trials, *Crocus sativus* demonstrated similar efficacy and a better safety profile compared to donepezil or memantine (Akhondzadeh *et al*., 2010; Farokhnia *et al*., 2014b). *Ginkgo biloba* extract (EGb 761) was also well tolerated, and its use was not associated with (serious) adverse events (van Dongen *et al*., 2000; Mazza *et al*., 2006). Furthermore, substances such as *Melissa* and *Salvia* have a direct positive effect on neuropsychiatric symptoms (BPSD), including a reduction in agitation and irritability, with agitation being less frequently observed in the groups treated with these herbs than in the placebo groups (Akhondzadeh *et al*., 2003a, 2003b).

Many of the clinical trials analysed, especially those involving new or less frequently studied substances, are characterised by significant limitations in sample size. Examples include studies with very small sample sizes, such as the *Cistanche tubulosa* study and *Ginkgo biloba* study, which included only 18 patients each (Le Bars *et al*., 1997; Guo *et al*., 2013), the *Melissa officinalis* study (23 participants) (Noguchi-Shinohara *et al*., 2020), *Choto-san* (20 participants) (Yamaguchi *et al*., 2004), *Yokukansan* (12 participants) (Nagata *et al*., 2012), ferulic acid and *Angelica archangelica* (20 participants) (Kimura *et al*., 2011), or the grape powder formulation study (10 participants) (Lee *et al*., 2017). This small sample size means that conclusions drawn from even large effect sizes (Cohen’s *d*), such as those observed in small studies with *Vitis vinifera* (*d* = 1.47) (Lee *et al*., 2017) or *Zataria multiflora* (*d* = 1.6) (Ansari *et al*., 2025), are subject to a high risk of bias. Small sample sizes make it difficult to assess whether the obtained large effect sizes (Cohen’s *d*) are reliable and whether a given phytopharmaceutical could actually be considered a disease-modifying drug (DMT).

The lack of standardisation of plant extracts is one of the most serious challenges in phytotherapy, which is directly reflected in the sources analysed. The use of different extraction methods and preparation forms (standardised extracts, powders, decoctions, concentrates) significantly contributes to the inconsistency of reported results and hinders their direct comparison. The analysed studies use dramatically different formulations, which directly translates into variability in results. For example, one study on *Melissa officinalis* used an extract standardised for rosmarinic acid content, achieving a medium effect size (*d* = −0.55) (Noguchi-Shinohara *et al*., 2020). However, an older study, which described the preparation only as ‘extract’, reported an extremely high effect size (*d* = −7.72) (Akhondzadeh *et al*., 2003a). This discrepancy may be due to differences in the concentration of active ingredients obtained using different extraction methods. Studies on *Panax ginseng* and *Cosmos caudatus* used powder, while studies on *Morus nigra* used concentrate (Lee *et al*., 2017; You *et al.*, 2021b; Güçer Öz *et al*., 2024). These formulations typically have lower compositional consistency than solvent-based extracts, which may explain why in some cases (e.g. ginseng) no difference was observed compared to placebo. The high heterogeneity index values calculated in our study (*I* ^2^ from 88.3% to 98.2%) provide mathematical evidence that the differences in results are not accidental. They result largely from the fact that different chemicals were actually tested in different studies under the same plant name (e.g. saffron or ginkgo) in terms of their phytochemical profile.

The clinical efficacy of phytopharmaceuticals must always be considered in the context of their safety, especially in the geriatric population, which often has multiple comorbidities. For instance, *Ginkgo biloba*’s phytocompounds (gingkolides) may inhibit platelet-activating factor (PAF). Therefore, patients taking anticoagulants (e.g. warfarin) or antiplatelet drugs (e.g. aspirin) should be monitored for an increased risk of bleeding, which is particularly important in older adults with cardiovascular disease (Bent *et al*., 2005; Koch, 2005). The lack of long-term studies on the toxicity and interactions of these extracts after many months of use requires caution in recommending them as stand-alone therapies in advanced stages of AD. Furthermore, many of the substances analysed, such as *Crocus sativus*, *Melissa officinalis*, *Salvia officinalis*, and *Bacopa monnieri*, affect the cholinergic pathway by inhibiting acetylcholinesterase (AChE) activity or receptor activity. Using them as a complementary option alongside standard medications, such as donepezil, may lead to additive pharmacodynamic effects. Although phytopharmaceuticals are often better tolerated than conventional medications, the lack of long-term studies on their use makes it difficult to fully assess the risk of cumulative adverse effects in complex drug regimens.

Another significant limitation is the short duration of interventions. Although some studies, such as those involving *Crocus sativus*, lasted up to 12 months (Farokhnia *et al*., 2014a; Tsolaki *et al*., 2016), a significant number of interventional studies lasted only 3 to 6 months, and the shortest only 4 weeks (Kimura *et al*., 2011; Iwasaki *et al*., 2012; Nagata *et al*., 2012 Albertyn *et al.,* 2025). Such a short time frame may be insufficient for evaluating phytotherapeutics as disease-modifying treatments (DMTs). We strongly recommend long-term follow-up periods (e.g. 12–24 months) for future research designs.

## Summary

In light of the evidence, phytopharmaceuticals have a promising role as a co-therapeutic option or alternative for patients with dementia who do not tolerate or have contraindications to standard medications. However, further research is necessary to translate these initial promising results into clinical practice. Longer-term, multicenter, randomised controlled trials (RCTs) with larger patient groups using consistent and sensitive outcome measures (such as the ADAS-Cog and NPI) are recommended. This is particularly important for compounds with large effects (e.g. *Zataria multiflora* and *Cosmos caudatus*), for which preliminary conclusions are limited by very small sample sizes.

Given that dementia represents a growing public health challenge, this area offers a critical, extensive, and high-priority scope for exploring novel therapeutic avenues.

## Supporting information

10.1017/neu.2026.10085.sm001 Kaczmarek-Kryszak et al. supplementary material 1 Kaczmarek-Kryszak et al. supplementary material

10.1017/neu.2026.10085.sm002 Kaczmarek-Kryszak et al. supplementary material 2 Kaczmarek-Kryszak et al. supplementary material

10.1017/neu.2026.10085.sm003 Kaczmarek-Kryszak et al. supplementary material 3 Kaczmarek-Kryszak et al. supplementary material

## Data Availability

All data collected for this systematic review are available in the text.

## Supplementary material

The supplementary material for this article can be found at https://doi.org/10.1017/neu.2026.10085.

## Registration

This systematic review was registered in the PROSPERO register under the number CRD420251239096.

## Figure 1. Long description

The diagram compares the molecular mechanisms of Alzheimer’s treatments Donepezil and Memantine. On the left, Donepezil enhances the cholinergic system through reversible AChE inhibition, increasing acetylcholine in the synaptic cleft, stimulating postsynaptic receptors, blocking the peripheral anionic site, exhibiting sigma-1 receptor agonist activity, and reducing amyloid beta levels and inflammation. On the right, Memantine regulates glutamatergic excitotoxicity through noncompetitive NMDA receptor antagonism, reducing excitotoxicity, exhibiting 5-HT3 and alpha7 nicotinic receptor antagonism, facilitating long-term potentiation, and reducing amyloid beta levels and inflammation.

Navigate back to Figure 1..

## Figure 2. Long description

Flowchart illustrating the stages of identifying studies via databases and registers. The process begins with records identified from databases such as PubMed, Web of Science, Embase, and registers, totaling 3059 records. Duplicate records, amounting to 790, are removed before screening. No records are marked as ineligible by automation tools, and three records are removed for other reasons. The remaining 2462 records are screened, with 2358 records excluded. Reports sought for retrieval total 82, with 11 reports not retrieved. Reports assessed for eligibility number 71, with exclusions due to language, multiple publications, administration methods, inconsistent study population, inconsistent studied parameters, and other reasons. Finally, 48 studies are included in the review.

Navigate back to Figure 2..

## Table 1. Long description

The table presents a descriptive synthesis of single herb interventions across various studies. It includes columns for the herb name, study details such as the number of participants and duration, and the results of the interventions. The table has multiple rows, each representing a different herb and its associated study data. Notable trends include the variety of herbs studied and the different outcomes measured in each study. The data type includes numerical values and descriptive text, providing a comprehensive overview of the interventions’ effectiveness.

Navigate back to Table 1..

## Table 2. Long description

The table presents a detailed synthesis of 48 studies on polyherbal interventions for cognitive functions, with 27 studies included in the statistical analysis of effect size. The table includes columns for author, year, study type, aim, main outcome measure, result, conclusion, study group, number of participants, sex of participants, age of participants, and study duration. Each row provides specific details for each study, such as the author’s name, the year of publication, the type of study conducted, the aim of the study, the main outcome measure used, the results obtained, the conclusions drawn, the study group, the number of participants, the sex of the participants, the age of the participants, and the duration of the study.

Navigate back to Table 2..

## Table 3. Long description

The table presents a systematic review of 48 studies, with 27 included in the statistical analysis of effect size. The studies focus on dementia and mild cognitive impairment, using diagnostic tests such as ADAS-cog and MMSE. The table includes columns for the author, year, condition, intervention, comparator, outcome, study design, mean change, standard deviation change, and effect size. Notable trends include the use of different diagnostic tests and the exclusion of studies with missing numerical data. Heterogeneity analysis was performed for plants included in more than one study.

Navigate back to Table 3..

## Table 4. Long description

The table presents a heterogeneity analysis of different plants versus placebo, focusing on the number of studies, Cochran’s Q, I squared percentage, and interpretation. It includes data for Ginkgo biloba, Crocus sativus, and Melissa officinalis. Each row lists the plant name, number of studies, Cochran’s Q value with p-value, I squared percentage, and an interpretation of the results. The table highlights the sources of inconsistency and the dramatic differences between studies for each plant.

Navigate back to Table 4..

## Table 5. Long description

The table presents the optimal doses of various plant extracts used in interventions and their mechanisms of action. It includes seven rows and three columns. The columns are labeled Plant, Optimal dose, and Mechanism of action. The plants listed are Ginkgo biloba, Crocus sativus, Bacopa monnieri, Melissa officinalis, Salvia officinalis, Panax ginseng, and Cosmos caudatus. The optimal doses range from 15 milligrams twice per day to 4.5 grams once per day. The mechanisms of action include antioxidant properties, iron chelation, GSH synthesis enhancement, AChE inhibition, NMDA antagonism, Aβ and tau antiaggregation, ChAT activation, neuronal arborisation increment, ACh pathway modulation, Aβ antiaggregation, axons and synapses generation, NPCs proliferation and differentiation.

Navigate back to Table 5..
