# Supplementary material for: A comprehensive systematic review of human trials investigating herbal treatments for Alzheimer’s disease and dementia
Source: Acta Neuropsychiatr. 2026 May 8;38:e42. doi: 10.1017/neu.2026.10085 (PMC13280518; doi:10.1017/neu.2026.10085)
Supplement: Kaczmarek-Kryszak et al. supplementary material 3 — Kaczmarek-Kryszak et al. supplementary material [file S0924270826100854sup003.pdf]

NIHR | National Institute for Health and Care Research

PROSPERO
International prospective register of systematic reviews

A comprehensive systematic review of human trials investigating herbal treatments for Alzheimer's disease and dementia

Katarzyna A. Kaczmarek-Kryszak, Małgorzata Dobrzyńska

Citation
Katarzyna A. Kaczmarek-Kryszak, Małgorzata Dobrzyńska. A comprehensive systematic review of human trials investigating herbal treatments for Alzheimer's disease and dementia . PROSPERO 2025 CRD420251239096. Available from https://www.crd.york.ac.uk/PROSPERO/view/CRD420251239096.

REVIEW TITLE AND BASIC DETAILS

Review title

A comprehensive systematic review of human trials investigating herbal treatments for Alzheimer's disease and dementia

Condition or domain being studied

Dementia; Alzheimer's Disease; Phytotherapy; Herbal medicine

Rationale for the review

Despite the growing number of clinical trials evaluating natural remedies, a thorough synthesis of the results is necessary to assess their true place in therapeutic regimens for Alzheimer's, vascular dementia, and mild cognitive impairment. The primary goal of this review is to comprehensively analyze the results of clinical trials and other human studies regarding the efficacy and safety of natural remedies and complex herbal formulas used in patients with dementia.

Review objectives

What is the effectiveness of herbal medications compared to standard treatment (donepezil, memantine) and placebo, the consistency of effects across different types of dementia, and aspects of the greatest clinical benefits of herbal medications?

Keywords

Alzheimer's disease; Demetia; Herbal medicine; Phytotherapy; Traditional Chinese medicine

Country

Poland

ELIGIBILITY CRITERIA

Population

Included
adult patients diagnosed with cognitive impairment - Alzheimer's disease (AD): Mild, moderate, or severe, vascular dementia (VaD) or mixed dementia, mild cognitive impairment (MCI), including vascular MCI and MCI associated with AD, other dementias

Excluded
healthy subjects or major comorbidities

Intervention(s) or exposure(s)

Included
Phytotherapy; Herbal medicine; Traditional Chinese Medicine

Phytopharmaceuticals and TCM: Herbal extracts or complex formulas.

Excluded
administration other than oral, intervention including phytotherapeutic and other treatments (standard drugs, cognitive training, physical activity)

Comparator(s) or control(s)

Included
PICO tags selected: Placebo; Active control

Studies had to include a control group (placebo) or an active comparator (e.g., donepezil, memantine, Ginkgo biloba*). In the absence of a control group, studies had to include paired measurements - before and after the intervention.

* Ginkgo biloba was used as a control intervention in some studies, as it is currently the best-researched phytotherapeutic, with relatively predictable and stable outcomes.

Study design

Both randomized and nonrandomized study types will be included.

Included
Randomized controlled trials (RCTs), double-blind, placebo-controlled studies, and, where

necessary, other clinical trials (e.g., open-label, uncontrolled) were included.

Context
We searched for publications concerning phytopharmaceutical interventions in patients suffering from different types of dementia. The publications had to include an oral administration of a phytocompound with either comparator or baseline/endpoint measurments `to asses cognitive function, functioning of daily living, behavioral and psychological symptoms of dementia, changes on the Neuropsychiatric Inventory (NPI) and NPI-Q scales, safety and tolerability.

TIMELINE OF THE REVIEW

Date of first submission to PROSPERO
24 November 2025

Review timeline
Start date: 1 September 2025. End date: 23 December 2025.

Date of registration in PROSPERO
25 November 2025

AVAILABILITY OF FULL PROTOCOL

Availability of full protocol
A full protocol has been written but is not available because:

It will be included in a published review.

SEARCHING AND SCREENING

Search for unpublished studies
Only published studies will be sought.

Main bibliographic databases that will be searched
The main databases to be searched are *CENTRAL - Cochrane Central Register of Controlled Trials*, *Embase.com*, *MEDLINE* and *PubMed*.

Other important or specialist databases that will be searched
Web of Science

Search language restrictions
The review will only include studies published in English.

Search date restrictions
There are no search date restrictions.

Other methods of identifying studies

Other studies will be identified by: *reference list checking (backward citation searching)* and *searching trial or study registers*.

Link to search strategy

A full search strategy has been uploaded to PROSPERO. The PDF may be accessed through this link https://www.crd.york.ac.uk/PROSPEROFILES/793659ed9f4aeeebec9cac3986f5c9c6.pdf.

Selection process

Studies will be screened independently by at least two people (or person/machine combination) with a process to resolve differences.

Other relevant information about searching and screening

None

DATA COLLECTION PROCESS

Data extraction from published articles and reports

Data will be extracted independently by at least two people (or person/machine combination) with a process to resolve differences.

Authors will not be contacted for further information.

Study risk of bias or quality assessment

Risk of bias will be assessed using:

For the risk of bias assessment, two researchers worked independently. We analyzed each of the research projects, the presence of a control group/control measurements, the use of validated tests.

Data will be assessed independently by at least two people (or person/machine combination) with a process to resolve differences.

Additional information will be sought from study investigators if required information is unclear or unavailable in the study publications/reports.

Reporting bias assessment

Risk of bias due to missing results will be assessed

Certainty assessment

Certainty of findings will not be assessed

OUTCOMES TO BE ANALYSED

Main outcomes

the efficacy and safety of natural remedies and complex herbal formulas used in patients with dementia

Additional outcomes

the consistency of effects across different types of dementia, and aspects of the greatest clinical benefits of herbal medications

PLANNED DATA SYNTHESIS

Strategy for data synthesis

Comparative analysis of the studies will be conducted based on p-value and effect size (Cohen's d). Effect sizes will be calculated based on the numerical values provided in the publication. In the absence of data in the text, we will use average values in this type of tests to calculate missing data (for ex. SD change).

CURRENT REVIEW STAGE

Stage of the review at this submission

Review stage	Started	Completed
Pilot work	✓	✓
Formal searching/study identification	✓	✓
Screening search results against inclusion criteria	✓	✓
Data extraction or receipt of IPD		
Risk of bias/quality assessment		
Data synthesis		

Review status

The review is currently planned or ongoing.

Publication of review results

Results of the review will be published in English.

REVIEW AFFILIATION, FUNDING AND PEER REVIEW

Review team members

Mrs Katarzyna A. Kaczmarek-Kryszak (review guarantor and contact) ORCID: 0009-0009-9329-6317. Poznan University of Medical Sciences. Poland.

No conflict of interest declared.

Dr Małgorzata Dobrzyńska. ORCID: 0000-0001-9589-522X. Poznan University of Medical

Sciences. Poland.

No conflict of interest declared.

Named contact

Mrs Katarzyna A. Kaczmarek-Kryszak (katarzynakaczmarek.official@gmai.com). ORCID: 0009-0009-9329-6317. Poznan University of Medical Sciences. Poland.

Review affiliation

Poznan University of Medical Sciences

Funding source

Review has no specific/external funding but is supported by guarantor/review team (non-commercial) institutions.

Additional information about funding
Paid doctoral scholarship

Peer review

There has been no peer review of this planned review.

ADDITIONAL INFORMATION

Review conflict of interest

Declared individual interests are recorded under team member details.. No additional interests are recorded for this review.

Medical Subject Headings

Alzheimer Disease; Humans; Phytotherapy; Herbal Medicine; Medicine, Chinese Traditional; Donepezil; Memantine

SIMILAR REVIEWS

Check for similar records already in PROSPERO

PROSPERO identified a number of existing PROSPERO records that were similar to this one (last check made on 24 November 2025). These are shown below along with the reasons given by that the review team for the reviews being different and/or proceeding.

- Comparative Safety and Effectiveness of Various Acetylcholinesterase Inhibitors and Memantine in Various Dementia Diagnoses: A Network Meta-Analysis [published 15 October 2024] [CRD42024597231]. The review was judged **not to be similar**
- An updated meta-analysis on the treatment effects between monotherapy of donepezil versus combination with memantine for Alzheimer's disease [published 26 April 2024] [CRD42024536151]. The review was judged **not to be similar**
- Effectiveness and safety comparisons of Chinese herbal medicines for Alzheimer’s

disease: a Bayesian network meta-analysis [published 28 April 2020] [CRD42020167155]. The review was judged **not to be similar**

PROSPERO version history

- Version 1.0, published 25 Nov 2025

Disclaimer

The content of this record displays the information provided by the review team. PROSPERO does not peer review registration records or endorse their content.

PROSPERO accepts and posts the information provided in good faith; responsibility for record content rests with the review team. The guarantor for this record has affirmed that the information provided is truthful and that they understand that deliberate provision of inaccurate information may be construed as scientific misconduct.

PROSPERO does not accept any liability for the content provided in this record or for its use. Readers use the information provided in this record at their own risk.

Any enquiries about the record should be referred to the named review contact
